# Association between fatty acid metabolism in the brain and Alzheimer disease neuropathology and cognitive performance: A nontargeted metabolomic study

**DOI:** 10.1371/journal.pmed.1002266

**Published:** 2017-03-21

**Authors:** Stuart G. Snowden, Amera A. Ebshiana, Abdul Hye, Yang An, Olga Pletnikova, Richard O’Brien, John Troncoso, Cristina Legido-Quigley, Madhav Thambisetty

**Affiliations:** 1 Institute of Pharmaceutical Science, King’s College London, London, United Kingdom; 2 Institute of Psychiatry, Psychology and Neuroscience, King’s College London, London, United Kingdom; 3 Laboratory of Behavioral Neuroscience, National Institute on Aging, Baltimore, Maryland, United States of America; 4 Division of Neuropathology, Johns Hopkins School of Medicine, Johns Hopkins University, Baltimore, Maryland, United States of America; 5 Department of Neurology, Duke University Medical School, Duke University, Durham, North Carolina, United States of America; 6 Clinical and Translational Neuroscience Unit, Laboratory of Behavioral Neuroscience, National Institute on Aging, Baltimore, Maryland, United States of America; University of Cambridge, UNITED KINGDOM

## Abstract

**Background:**

The metabolic basis of Alzheimer disease (AD) pathology and expression of AD symptoms is poorly understood. Omega-3 and -6 fatty acids have previously been linked to both protective and pathogenic effects in AD. However, to date little is known about how the abundance of these species is affected by differing levels of disease pathology in the brain.

**Methods and findings:**

We performed metabolic profiling on brain tissue samples from 43 individuals ranging in age from 57 to 95 y old who were stratified into three groups: AD (*N* = 14), controls (*N* = 14) and “asymptomatic Alzheimer’s disease” (ASYMAD), i.e., individuals with significant AD neuropathology at death but without evidence for cognitive impairment during life (*N* = 15) from the autopsy sample of the Baltimore Longitudinal Study of Aging (BLSA). We measured 4,897 metabolite features in regions both vulnerable in the middle frontal and inferior temporal gyri (MFG and ITG) and resistant (cerebellum) to classical AD pathology. The levels of six unsaturated fatty acids (UFAs) in whole brain were compared in controls versus AD, and the differences were as follows: linoleic acid (*p* = 8.8 x 10^−8^, FC = 0.52, q = 1.03 x 10^−6^), linolenic acid (*p* = 2.5 x 10^−4^, FC = 0.84, q = 4.03 x 10^−4^), docosahexaenoic acid (*p* = 1.7 x 10^−7^, FC = 1.45, q = 1.24 x 10^−6^), eicosapentaenoic acid (*p* = 4.4 x 10^−4^, FC = 0.16, q = 6.48 x 10^−4^), oleic acid (*p* = 3.3 x 10^−7^, FC = 0.34, q = 1.46 x 10^−6^), and arachidonic acid (*p* = 2.98 x 10^−5^, FC = 0.75, q = 7.95 x 10^−5^). These fatty acids were strongly associated with AD when comparing the groups in the MFG and ITG, respectively: linoleic acid (*p* < 0.0001, *p* = 0.0006), linolenic acid (*p* < 0.0001, *p* = 0.002), docosahexaenoic acid (*p* < 0.0001, *p* = 0.0024), eicosapentaenoic acid (*p* = 0.0002, *p* = 0.0008), oleic acid (*p* < 0.0001, *p* = 0.0003), and arachidonic acid (*p* = 0.0001, *p* = 0.001). Significant associations were also observed between the abundance of these UFAs with neuritic plaque and neurofibrillary tangle burden as well as domain-specific cognitive performance assessed during life. Based on the regional pattern of differences in brain tissue levels of these metabolites, we propose that alterations in UFA metabolism represent both global metabolic perturbations in AD as well as those related to specific features of AD pathology. Within the middle frontal gyrus, decrements in linoleic acid, linolenic acid, and arachidonic acid (control>ASYMAD>AD) and increases in docosahexanoic acid (AD>ASYMAD>control) may represent regionally specific threshold levels of these metabolites beyond which the accumulation of AD pathology triggers the expression of clinical symptoms. The main limitation of this study is the relatively small sample size. There are few cohorts with extensive longitudinal cognitive assessments during life and detailed neuropathological assessments at death, such as the BLSA

**Conclusions:**

The findings of this study suggest that unsaturated fatty acid metabolism is significantly dysregulated in the brains of patients with varying degrees of Alzheimer pathology.

## Abstract 2

In a nontargeted metabolomic study, Cristina Legido-Quigley and colleagues investigate potential associations between fatty acid levels in the brain and Alzheimer disease neuropathology and cognitive performance.

## Author summary

**Why was this study done?:**

- Currently it is thought that the main cause of memory problems in association with dementia is the presence of two big molecules in the brain called tau and amyloid proteins.
- The small molecule changes that occur inside the brain during Alzheimer disease are unknown.

**What did the researchers do and find?:**

- We compared the differences in hundreds of small molecules in the brain in three groups: 14 people with healthy brains, 15 that had tau and amyloid but didn’t show memory problems, and 14 that had these proteins and had memory loss.
- Then we also had a look at three different areas in the brain, one that usually shows no tau or amyloid deposition, one that shows more tau, and another that shows more amyloid.
- The main molecules that were different were six small fats, such as omegas, which were different everywhere in the brain.

**What do these findings mean?:**

- Each fat showed a specific trend in Alzheimer patients.
- The number of people in the study was small, but the results suggest a role of fats in dementia.

## Introduction

Alzheimer disease (AD) is a neurodegenerative disorder characterised by progressive cognitive decline, with impairment in multiple cognitive domains including memory, executive function, and language [1]. AD accounts for between 60% and 80% of total dementia cases worldwide [2] and represents a major cause of global morbidity and mortality. It is currently estimated that there are over 46 million people suffering from the disease worldwide, with the number of patients estimated to rise to 131.5 million by 2050 [3]. As well as a major human cost, dementia also imposes a significant economic impact costing US$818 billion in 2015, and estimated to rise to US$1 trillion by 2018 [3].

Extracellular accumulation of amyloid-β (Aβ) plaques and intracellular accumulation of neurofibrillary tangles are the pathological hallmarks of AD. A growing body of evidence suggests that metabolic perturbations in various pathways may mediate the occurrence of Alzheimer pathology as well as the onset of cognitive impairment in patients [4]. The application of large-scale unbiased metabolomics techniques to study the role of metabolism in Alzheimer pathogenesis may facilitate a more complete understanding of Alzheimer pathology and mechanisms triggering symptom expression. Metabolomics can be defined as “the unbiased analysis of the composition of small molecule metabolites in a given biological tissue or fluid under a specific set of environmental conditions” [5–7]. Several metabolomic studies have previously examined the relationship between metabolism and AD pathology. While studies on serum, plasma, and cerebrospinal fluid have identified several metabolic pathways involving bile acids, sphingolipids, antioxidants, phospholipids, and amino acids that appear to be associated with disease [8–13], it is likely that metabolomic studies on human brain tissue samples may provide direct insights into the molecular basis of Alzheimer pathogenesis. A handful of studies have performed metabolomics on brain tissue samples from both transgenic animal models and humans. Salek et al. found alterations in neurotransmitters, amino acids and antioxidants to be strongly associated with AD [14]. Graham et al. [15] used metabolomics to analyze human brain neocortex samples (15 controls versus 15 AD) and were able to differentiate controls and AD samples, [15]. Inoue et al. analyzed the parietal and frontal lobes of healthy controls and Alzheimer patients (10 versus 10) and identified increased brain polyamine metabolism in AD brains [16].

In AD, there is a regional specificity in the vulnerability of various brain regions to pathology, with some regions exhibiting greater amyloid pathology whilst others are dominated by neurofibrillary tangles [17,18]. However, previous studies have not examined changes in brain metabolite profiles in relation to the regional distribution of disease pathology. Equally important, few studies have addressed the relationships between brain metabolite signatures and the manifestation of clinical symptoms of AD in response to pathology. It is now well recognized that substantial levels of Alzheimer pathology can occur in the brains of cognitively normal individuals [19]. In the Baltimore Longitudinal Study of Aging (BLSA), we have categorized this group of individuals as “asymptomatic Alzheimer’s disease” (ASYMAD) [20], i.e., subjects with significant AD neuropathology at death but without evidence for cognitive impairment during life, as assessed by longitudinal cognitive assessments [20].

In this study, we applied untargeted metabolomics to brain tissue collected through the autopsy sample of the BLSA in three groups of individuals (AD; *N* = 14, control; *N* = 14 and ASYMAD; *N* = 15) and studied differences in brain metabolite levels within regions both vulnerable and resistant to AD pathology. We thus examined the middle frontal gyrus (MFG; vulnerable to Aβ deposition), inferior temporal gyrus (ITG; vulnerable to tau deposition), and the cerebellum (CB), which is relatively spared of classical AD pathology [21].

The relationships between metabolic shifts and specific features of Alzheimer pathology and disease outcome were assessed by applying untargeted metabolomics utilizing liquid chromatography–mass spectrometry (LC-MS) and gas chromatography–mass spectrometry (GC-MS) to maximize the metabolite coverage obtained.

## Materials and methods

### Sample information

The ethics approval was obtained from the Institutional Review Board of the University of Maryland Baltimore County (03-AG-0325 THE BALTIMORE LONGITUDINAL STUDY OF AGING). The BLSA is a prospective, ongoing cohort study of community-dwelling volunteer participants in Baltimore that originated in 1958. As such, it is among the largest and longest running longitudinal studies of aging in the United States [22,23]. In general, at the time of entry into the study, participants had no physical or cognitive impairment. Detailed examinations, including neuropsychological assessments and neurological, laboratory, and radiological evaluations, were conducted every 2 y. Since 2003, participants older than 80 y have received yearly assessments. Written informed consent was obtained at each visit, and the study was approved by the local Institutional Review Board and the National Institute on Aging. After each visit, cognitive status was considered at consensus diagnosis conferences relying on information from neuropsychological tests as well as clinical data as described previously [24]. Diagnoses of dementia and AD were based on DSM-III-R [25] and the NINCDS-ADRDA criteria [26], respectively.

Brain tissue was collected through the autopsy sample of the BLSA. The autopsy program of the BLSA was initiated in 1986. We have previously described the study protocol in detail. Briefly, the mean age at death in the autopsy sample is 88.3 ± 7.3 y (range 69.3–103.2), and the mean interval between last evaluation and death is 8.7 ± 6.7 mo [27]. As reported previously, the autopsy subsample is not significantly different from the BLSA cohort as a whole in terms of the rates of dementia and clinical stroke [28]. Table 1 describes the demographic characteristics of the participants whose brain tissue samples were used in this study.

**Table 1:** Clinical characteristics of study participants.

	Control	Asymptomatic	AD
Participants (f/m)	14 (4/10)	15 (5/10)	14 (7/7)
Age at death (y)^a^	82.6 +/− 11.0 (64.2–99.2)	89.2 +/− 7.9 (71.9–96.4)	87.9 +/− 8.9 (62.9–98.7)
MMSE^b^	27.8 +/− 2.4 (23–30)	29.0 +/− 0.9* (25–30)	23.0 +/− 6.9** (8–30)
PMI (h) ^c^	16.9 +/− 6.4 (7.0–28.0)	14.8 +/− 8.1 (2.0–33.0)	14.7 +/− 6.0 (3.0–23.0)
ApoE^d^	5/7/1/1	1/10/4/0	3/7/3/1

^a^ values are reported as the mean +/− standard deviation.

^b^ range values are reported as the mean +/− standard deviation.

^c^ values are reported as the mean +/− standard deviation.

^d^ range distribution of ApoE genotypes (e2:e3/e3:e3/e3:e4/e4:e4). No participants were e2:e2 or e2:e4.

MMSE: Mini-mental state examination, PMI: postmortem interval.

*p<0.05.

**p<0.01.

The calculated p-values were relative to controls.

### Chemicals and reagents

All solvents and reagents, water, methanol, acetonitrile, ammonium formate, formic acid, and methyl tertiary butyl ether (MTBE), were LC-MS grade purchased from Sigma-Aldrich. Two internal standards for LC-MS analysis were added, L-serine^13^C_3_^15^N (95%) and L-valine^13^C_5_^15^N (95%), purchased from Sigma-Aldrich Sample derivatization for GC-MS analysis was performed using N,O-Bis(trimethylsilyl)trifluoro-acetamide (BSTFA) with 1% trimethylchlorosilane (TMCS) purchased from Sigma-Aldrich.

### Sample preparation

The in-vial dual extractions were performed as previously described [7]. After LC-MS analysis, the remaining aqueous and nonaqueous phases were split into separate vials and dried down under a stream of nitrogen at 37°C. Samples were then resuspended in a 1:1 solution of acetonitrile and the derivatizing agents N,O-Bis(Trimethylsilyl)trifluoroacetamide and 1% Trimethylchlorosilane, and were incubated at 37°C for 1 h. After incubation, samples were again dried down under nitrogen at 37°C and were subsequently resuspended in 25 μl of toluene for analysis.

### Metabolite acquisition

Samples were analysed using HILIC LC-MS analysis performed as described previously [7]. GC-MS analysis was carried out on a Shimadzu QP-2010 with an AOC-20S autosampler and AOC-20i autoinjector (Shimadzu, Kyoto, Japan). The aqueous phase was analysed in split less mode with 2 μl of sample injected on a BP5MS capillary column (length 30 m, thickness 0.25 mm, diameter 0.25 mm). The carrier gas (helium) pressure was 79.5 Kpa, with a total flow of 125 ml/min, a column flow of 1.18 ml/min, a linear velocity of 40 cm/s, and a purge flow of 6 ml/min. The gradient temperature started at 80°C and was held for 5 min followed by a linear increase of 10°C per min to 200°C, where the rate of increase was slowed to 2°C per min to a final temperature of 225°C, and was held for 4 min. Analysis of 1 μl nonaqueous phase was performed in the split less mode on the same column. The carrier gas (helium) pressure was set to 86.2 Kpa with a total flow of 122.8ml/min, a column flow of 1.16 ml/min, a linear velocity of 40cm/s, and a purge flow of 6 ml/min. The gradient temperature started at 100°C and was held for 5 min, followed by a linear increase of 15°C per min to 250°C, where the rate of increase was slowed to 2°C per min to a final temperature of 310°C, where it was held for 4 min. Mass spectral analysis of both phases was performed using electron ionisation between 50 and 600 m/z with an ion source temperature of 200°C, an interface temperature of 280°C with a scan speed of 833, and an event time of 0.2 s.

### Data processing

Initially, all raw data files were converted into.mzXML format, LC-MS files were converted using msConvert (ProteoWizard) whilst GC-MS files were converted using GCMS Solutions (Shimadzu). Converted data files were analysed using XCMS and performed in the open source software package R, with peak picking performed on all MS assays using a “centwave” method, which allows the deconvolution of closely eluting or slightly overlapping peaks. After peak picking, metabolite features were defined as peaks with an average intensity 5 times higher in analytical samples than is measured in the extraction blanks. Metabolite features were analysed using a range of multivariate tests, with all of the data logarithmically transformed (base10) and scaled to unit variance (UV), including principal component analysis (PCA) and partial least square–discriminant analysis (PLS-DA) performed in SIMCA 13.0.4 (Umetrics, Umeå, Sweden). Model performance was assessed based on the cumulative correlation coefficients (R^2^X[cum]) and predictive performance based on 7-fold cross validation (Q^2^[cum]), with the significance of the model assessed based on the ANOVA of the cross-validated residuals (CV-ANOVA). Feature selection to create curated models was performed by iteratively removing metabolite features that had a score of less than 1 in the variable importance to projections plot to achieve the fitted model with the optimal R^2^ and Q^2^ values. All metabolite annotations were made by matching metabolite fragmentation patterns to those in both in-house and publicly available spectral libraries.

To compare the fatty acids among three groups (CN, ASYMAD, and DEMENT), we used nonparametric Kruskal–Wallis test for testing the null hypothesis that all three groups are equal and Mann–Whitney U test for pairwise comparisons. To control for type 1 errors in the *p*-values calculated using the Mann-Whitney U test, two false discovery rate strategies were employed. The first was to determine which *p*-values passed a Bonferroni corrected significance threshold and the second was to subject the *p*-values to a Benjamini-Hochberg procedure performed in “R.” The relationship of metabolite abundance to measures of neuritic plaque and neurofibrillary tangle burdens in the brain as described by the Consortium to Establish a Registry for Alzheimer’s Disease (CERAD) and Braak scores, respectively, were determined by calculating the Pearson’s product-moment correlation coefficient. To evaluate the effect of fatty acids on longitudinal cognitive performance, we created domain-specific composite scores. We standardized each of the cognitive measures (using their baseline mean and standard deviation) and averaged the corresponding Z scores to form the composite score. Trail making tests A and B were first log transformed and then standardized. The following five cognitive domains were created: memory is the composite score of CVLT, learning and immediate free recall and CVLT long delay free recall, attention is the composite score of Trail making test A and WAIS-R Digits Forward, executive function is the composite score of Trail making test B and WAIS-R Digits Backward, language is the composite score of Letter Fluency and Semantic Fluency, and visuospatial ability is the composite score of clock drawing and card rotation tests.

We then used separate linear mixed models with each cognitive domain as the outcome variable. The main predictor time (or time of follow up) was anchored at the last visit, and all the previous longitudinal follow-up visits were negative relative to the last visit. This kind of recentering of the time variable allows us to test the effect of the levels of each fatty acid on the cognitive performance at last visit and cognitive rates of change simultaneously in a single model. The predictors included fatty acid levels, sex, age at last visit, time, and interactions of time with fatty acid levels, sex, and age at last visit. Random effects included intercept and time with unstructured covariance. Sex was coded −0.5 for females and 0.5 for males, age at last visit was mean centered, each fatty acid level was standardized. All analyses were conducted in SAS 9.4 (Cary, NC).

## Results

The clinical characteristics of the three diagnostic groups analysed are summarised in Table 1. The three groups did not differ significantly in age at death, postmortem interval, sex, or APOE ε4 status. In this study, a total of 4,897 metabolite features were measured, 3,482 by LC-MS and 1,415 by GC-MS. Of these measured metabolite features, 126 were successfully annotated, representing 100 structurally distinct metabolites (S1 Table, S2 Table).

Multivariate models were constructed based on all 4,897 metabolite features to assess the effects of both brain region and pathological diagnosis on metabolite composition. In curated PLS-DA models, it can be seen that both brain region (S1 Fig: R^2^X = 0.639, R^2^Y = 0.622, Q^2^ = 0.608, cross validated ANOVA [CV-ANOVA] = 5.65 × 10^−33^) and pathological diagnosis (S1 Fig: R^2^X = 0.499, R^2^Y = 0.439, Q^2^ = 0.404, CV-ANOVA = 4.72 × 10^−21^) are significantly associated with metabolite composition. The PLS-DA scores plots (S1 Fig) show that all three of the brain regions possess unique metabolite compositions, whilst the MFG and the ITG are compositionally distinct, shown by a modest separation in the second component (t[2]), and are more similar to each other than they are with the CB (S1 Fig), which is highly compositionally distinct, as shown by a clear separation in the first component (t[1]). As with the brain regions, PLS-DA analysis showed that the three diagnostic groups possessed unique chemical compositions, with less difference observed between control and ASYMAD, shown by the modest separation in the second component, with the AD groups the most distinct with the greatest separation in the first component.

Having shown that AD pathology is significantly associated with brain metabolite levels, we then examined specific metabolites and metabolic pathways driving the observed shifts. The metabolite features that were used to generate the optimal curated PLS-DA models in all three diagnostic groups (S1 Fig) were taken forward for further analyses of their relationships to AD pathology and disease status. The optimal curated PLS-DA model was comprised (S1 Fig) of 876 metabolite features putatively representing 160 metabolites as associated with diagnostic status, with 31 of these metabolites annotated (Table 2).

**Table 2:** Annotated metabolites in order of significance (*p*-value) for the comparison of controls versus individuals with dementia.

Metabolite	Control versus Asymptomatic			Control versus Dementia		
	*p*-value	q-value^a^	FC^b^	*p*-value	q-value^a^	FC^b^
**Cholesterol**	7.5 × 10^−3^	4.96 × 10^−2^	0.74	6.8 × 10^−8^^+^	1.03 × 10^−6^	0.53
**Linoleic Acid**	7.3 × 10^−3^	5.81 × 10^−2^	0.74	8.8 × 10^−8^^+^	1.03 × 10^−6^	0.52
**Cholestenol**	1.0 × 10^−1^	5.81 × 10^−2^	1.17	1.0 × 10^−7^^+^	1.03 × 10^−6^	1.44
**Docosahexaenoic acid**	1.7 × 10^−1^	5.81 × 10^−2^	1.14	1.7 × 10^−7^^+^	1.24 × 10^−6^	1.45
**Carbamic acid**	1.9 × 10^−1^	8.68 × 10^−2^	1.15	2.0 × 10^−7^^+^	1.24 × 10^−6^	1.53
**Methylheptadecadiynoic acid**	2.4 × 10^−2^	1.50 × 10^−1^	0.79	2.8 × 10^−7^^+^	1.45 × 10^−6^	0.59
**Oleic acid**	5.5 × 10^−2^	1.73 × 10^−1^	0.73	3.3 × 10^−7*^^+^	1.46 × 10^−6^	0.34
**Palmitic acid**	1.9 × 10^−2^	1.73 × 10^−1^	0.73	4.8 × 10^−7*^^+^	1.86 × 10^−6^	0.44
**Hexanedioic acid**	2.7 × 10^−1^	1.73 × 10^−1^	1.11	6.4 × 10^−7^^+^	2.20 × 10^−6^	1.42
**Dimethylglycine**	2.9 × 10^−2^	1.73 × 10^−1^	0.89	1.0 × 10^−6^^+^	3.10 × 10^−6^	0.74
**Guanidobutanoate**	1.9 × 10^−1^	1.73 × 10^−1^	0.94	3.7 × 10^−6^^+^	1.04 × 10^−5^	0.75
**Ascorbate**	1.5 × 10^−1^	1.73 × 10^−1^	1.20	1.9 × 10^−5^^+^	4.91 × 10^−5^	1.54
**Aminobutanal**	6.7 × 10^−2^	1.79 × 10^−1^	0.94	9.1 × 10^−5^^+^	2.07 × 10^−4^	0.86
**Gluconic acid**	1.4 × 10^−2^	1.90 × 10^−1^	1.31	1.0 × 10^−4^^+^	2.07 × 10^−4^	1.51
**Cysteine**	4.7 × 10^−1^	1.90 × 10^−1^	1.06	1.0 × 10^−4^^+^	2.07 × 10^−4^	1.33
**Aspartate**	6.2 × 10^−2^	1.94 × 10^−1^	1.17	1.5 × 10^−4^^+^	2.91 × 10^−4^	0.84
**L-DOPA**	9.2 × 10^−2^	2.55 × 10^−1^	1.11	1.8 × 10^−4*^^+^	3.27 × 10^−4^	1.23
**Fumaric acid**	4.4 × 10^−2^	2.56 × 10^−1^	0.91	1.9 × 10^−4^^+^	3.27 × 10^−4^	0.85
**Linolenic acid**	7.5 × 10^−2^	2.56 × 10^−1^	0.90	2.5 × 10^−4^^+^	4.03 × 10^−4^	0.84
**Indoleacetic acid**	1.4 × 10^−1^	2.56 × 10^−1^	0.93	2.6 × 10^−4^^+^	4.03 × 10^−4^	0.82
**Eicosapentaenoic acid**	1.6 × 10^−3^	2.56 × 10^−1^	0.25	4.4 × 10^−4^	6.48 × 10^−4^	0.16
**Allantoin**	6.9 × 10^−3^	2.56 × 10^−1^	1.19	4.6 × 10^−4^	6.48 × 10^−4^	1.30
**Hypoxanthine**	4.7 × 10^−1^	2.56 × 10^−1^	0.98	5.4 × 10^−4^	7.28 × 10^−4^	0.88
**Coumaric acid**	5.9 × 10^−2^	3.10 × 10^−1^	1.16	7.6 × 10^−4^	9.82 × 10^−4^	1.28
**Adenine**	9.2 × 10^−2^	3.35 × 10^−1^	1.10	8.0 × 10^−4^	9.92 × 10^−4^	1.18
**Oxoarginine**	9.0 × 10^−1^	4.29 × 10^−1^	0.99	8.6 × 10^−4^	1.03 × 10^−3^	0.77
**Deoxyflurouridine**	5.2 × 10^−1^	5.20 × 10^−1^	1.08	1.0 × 10^−3^	1.15 × 10^−3^	1.45
**Arginine**	5.7 × 10^−2^	5.20 × 10^−1^	1.24	1.1 × 10^−3^	1.22 × 10^−3^	1.38
**GABA**	3.6 × 10^−1^	5.56 × 10^−1^	1.07	4.4 × 10^−3^	4.70 × 10^−3^	1.18
**Methylstearate**	5.4 × 10^−1^	5.58 × 10^−1^	0.85	2.6 × 10^−2^	2.69 × 10^−2^	0.56
**Octadecanal**	1.6 × 10^−1^	9.00 × 10^−1^	2.41	5.6 × 10^−1^	5.60 × 10^−1^	0.68

^a^ q-value calculated using Benjamini-Hochberg (0.05 threshold).

^b^ fold change in metabolite abundance relative to controls.

^+^ significant below Bonferroni correct *p*-value = 3.14 × 10−^4.^

DOPA: dihydroxy-phenylalanine, GABA: gamma-aminobutyrate.

These annotated metabolites were subsequently mapped onto known metabolic pathways using the Kyoto Encyclopedia of Genes and Genomes’ pathway mapping tool [29]. This approach identified 113 metabolic pathways that contained at least one of the 31 metabolites. Of these 113 metabolic pathways, poly-unsaturated fatty acid (PUFA) metabolism was identified for more detailed analysis, as the metabolites in this pathway showed the strongest association with diagnostic status.

**Fig 1:**
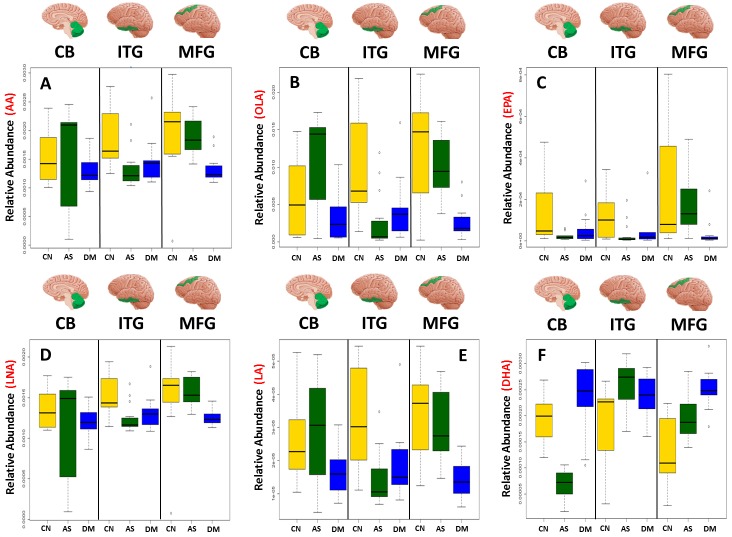
Boxplots showing the effect of disease status on the abundance of six UFAs in the CB, ITG, and MFG. Dysregulation of six UFAs shown by boxplots of three disease statuses separated by brain region A) arachidonic acid (AA), B) oleic acid (OLA), C) eicosapentaenoic acid (EPA), D) linolenic acid (LNA), E) linoleic acid (LA), F) docosahexaenoic acid (DHA). AS, asymptomatic Alzheimer; CN, control; DM, Dementia/Alzheimer.

Table 3 and Fig 1 summarize results of analyses comparing brain tissue unsaturated fatty acid (UFA) levels between the three groups. The overall pattern of results showed that brain tissue levels of the UFAs, linoleic acid, linolenic acid, eicosapentaenoic acid (EPA), oleic acid, and arachidonic acid were reduced in the ITG and MFG regions in AD relative to the control group. The AD group showed higher tissue levels of docosahexaenoic acid (DHA) in the ITG and MFG regions relative to controls. Within the CB, EPA levels also appeared to be lower in AD with oleic acid levels showing a similar trend relative to controls. Cerebellar DHA levels in AD were also higher relative to controls. Within the MFG, decrements in the levels of oleic acid, linoleic acid, linolenic acid, and arachidonic followed the pattern control>ASYMAD>AD. Increments in DHA levels in the MFG followed the pattern AD>ASYMAD>control.

**Table 3:** Relative differences in the abundance of six UFAs between all three diagnostic groups in individual brain regions.

Fatty acid	Overall *p*-value		Cont versus Asymp			Cont versus Dem			Asymp versus Dem		
	*p*-value^a^	q-value^c^	*p*-value^b^	q-value^c^	FC	*p*-value^b^	q-value^c^	FC	*p*-value^b^	q-value^c^	FC
**CB region**											
Linoleic	0.0910	0.1024									
Oleic	0.0160	0.0192	0.1800	0.3240	1.67	0.0840	0.1008	0.49	0.0089	0.0200	0.29
Docosahexanoic	<0.0001^+^	<0.0003	0.0003	0.0027	0.34	0.0330	0.0424	1.24	0.0001^+^	0.0004	3.67
Arachidonic	0.2900	0.2900									
Linolenic	0.2600	0.2753									
Eicosapentaenoic	0.0110	0.0141	0.0062	0.0140	0.11	0.0280	0.0388	0.27	0.3000	0.3600	2.32
**ITG region**											
Linoleic	0.0006	0.0012	0.0006	0.0027	0.42	0.0130	0.0234	0.57	0.0640	0.1152	1.34
Oleic	0.0003	0.0007	0.0006	0.0027	0.26	0.0043	0.0111	0.43	0.0340	0.0680	1.64
Docosahexanoic	0.0024	0.0033	0.0018	0.0054	1.43	0.0220	0.0330	1.30	0.1000	0.1500	0.91
Arachidonic	0.001	0.0016	0.0008	0.0029	0.71	0.0069	0.0155	0.77	0.2100	0.2700	1.09
Linolenic	0.002	0.0030	0.0021	0.0054	0.81	0.0081	0.0162	0.86	0.1800	0.2492	1.06
Eicosapentaenoic	0.0008	0.0014	0.0004	0.0027	0.09	0.0220	0.0330	0.21	0.0920	0.1500	2.24
**MFG region**											
Linoleic	<0.0001^+^	<0.0003	0.2900	0.4350	0.86	0.0001^+^	0.0009	0.41	<0.0001^+^	<0.00036	0.47
Oleic	<0.0001^+^	<0.0003	0.2000	0.3273	0.41	0.0004	0.0018	0.30	<0.0001^+^	<0.00036	0.73
Docosahexanoic	<0.0001^+^	<0.0003	0.0110	0.0220	1.25	<0.0001^+^	<0.0009	1.39	0.0037	0.0095	1.11
Arachidonic	0.0001^+^	0.0003	0.6100	0.8446	0.98	0.0009	0.0027	0.69	<0.0001^+^	<0.00036	0.69
Linolenic	<0.0001^+^	<0.0003	0.7100	0.9000	0.91	0.0004	0.0018	0.81	<0.0001^+^	<0.00036	0.90
Eicosapentaenoic	0.0002^+^	0.0005	0.7800	0.9000	1.25	0.0006	0.0022	0.08	0.0003^+^	0.0009	0.06

^a^ *p*-value calculated using Kruskal-Wallis test.

^b^ *p*-value calculated using Mann-Whitney U test.

^c^ q-value calculated using Benjamini-Hochberg (0.05 threshold).

^+^ significant below Bonferroni correct *p*-value = 3.14 × 10^−4^.

Asymp, asymptomatic AD; Cont, control; Dem, dementia AD; FC, fold change.

**Fig 2:**
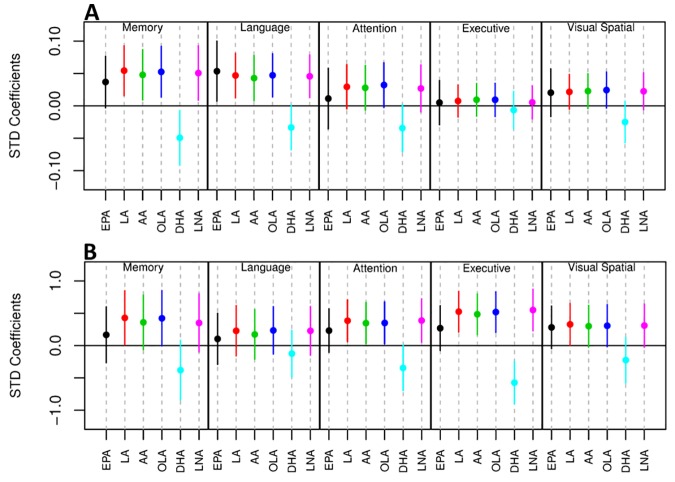
Forest plot showing the relationship of the abundance of six UFAs in the MFG and five measures of cognitive performance. **A)** Association between UFA levels and longitudinal cognitive performance. **B)** Association between UFA levels and cognitive performance at the last visit prior to death. AA, arachidonic acid; DHA, docosahexaenoic acid; EPA, eicosapentaenoic acid; LA, linoleic acid; LNA, linolenic acid; OLA, oleic acid; STD, Standard.

As well as associating with disease status, levels of all six UFAs in the MFG and ITG were shown to correlate significantly with both measures of neurofibrillary pathology estimated by Braak score and amyloid plaque burden assessed by the CERAD score (Table 4). Assessments of brain tissue levels of these UFAs in relation to domain-specific measures of cognitive performance showed consistent patterns across domains and several significant cross sectional and longitudinal associations across the three brain regions were found (S3–S7 Tables and S3 Fig and S4 Fig). In general, the pattern of these associations showed that lower tissue levels of linoleic acid, linolenic acid, EPA, oleic acid, and arachidonic acid were related to worse cognitive performance, whereas higher brain DHA levels were associated with poorer cognitive performance. The strongest associations with cognitive performance were between abundance of these fatty acids in the MFG and the final premortem memory, attention and executive function scores (Fig 2), as well as longitudinal trajectories in memory and language performance. The strongest association observed in the ITG between levels of these fatty acids was with longitudinal trajectory of visuospatial performance (S4 Fig).

**Table 4:** Correlation analysis of the abundance of UFAs and CERAD and Braak scores.

Region	Fatty acid	Braak (tau)		CERAD (Aβ)	
		r	*p*-value	r	*p*-value
**Cerebellum**	**EPA**	−0.31	0.096	−0.35	0.055
	**Linoleic**	−0.13	0.497	−0.19	0.306
	**Arachidonic acid**	−0.11	0.559	−**0.19**	**0.033**
	**Oleic acid**	−0.28	0.133	−0.27	0.142
	**DHA**	**0.41**	**0.024**	0.34	0.068
	**Linolenic acid**	−0.36	0.053	0.30	0.108
**ITG**	**EPA**	−**0.33**	**0.042**	−**0.36**	**0.028**
	**Linoleic**	−**0.38**	**0.018**	−**0.35**	**0.032**
	**Arachidonic acid**	−**0.38**	**0.020**	−**0.37**	**0.025**
	**Oleic acid**	−**0.34**	**0.040**	−**0.35**	**0.034**
	**DHA**	0.31	0.058	**0.32**	**0.048**
	**Linolenic acid**	−0.26	0.115	−0.32	0.052
**MFG**	**EPA**	−**0.61**	**<0.001**	−**0.53**	**<0.001**
	**Linoleic**	−**0.57**	**<0.001**	−**0.58**	**<0.001**
	**Arachidonic acid**	−**0.56**	**<0.001**	−**0.55**	**<0.001**
	**Oleic acid**	−**0.64**	**<0.001**	−**0.64**	**<0.001**
	**DHA**	**0.60**	**<0.001**	−**0.69**	**<0.001**
	**Linolenic acid**	−**0.63**	**<0.001**	−**0.67**	**<0.001**

Relationships between global measures of amyloid and tau pathologies and the regional abundances of six UFAs, values highlighted in bold are significant at *p* < 0.05.

## Discussion

The metabolic basis of vulnerability to AD pathology and the subsequent expression of symptoms of AD are poorly understood. In this study, we applied mass spectrometry-based metabolomics to human brain tissue samples from a well-characterized longitudinal cohort, i.e., the BLSA, to address this issue. Our results suggest that perturbations in brain UFA metabolism are closely related to AD pathogenesis. To the best of our knowledge, this report is the first to measure brain tissue levels of UFAs to demonstrate a relationship with both severity of AD pathology and the expression of AD symptoms. Including brain tissue samples from “ASYMAD” individuals who represent an intermediate group in the gradation of neuropathology from controls to AD patients in the absence of cognitive impairment during life allowed us to relate measures of brain UFAs to incremental levels of AD pathology and symptom expression. Importantly, by measuring UFA levels in brain regions both vulnerable to distinct pathological features of AD, i.e., MFG (amyloid deposition) and ITG (tau accumulation) as well as in a region relatively resistant to AD pathology, i.e., CB, we were able to ask whether the observed alterations in these metabolites were related to AD-defining pathological processes. We can categorize our results broadly as follows:

- In the CB, the observed shifts in the abundance of the six UFAs measured do not follow a consistent trend (Fig 1) as observed in both the ITG and MFG, which are vulnerable to tau and amyloid pathology, respectively. Consistent shifts in the abundance of the UFAs are observed in the ITG, with the greatest difference between control and ASYMAD participants (Fig 1). Consistent shifts are also observed in the MFG, with the ASYMAD group being intermediate (Control>ASYMAD>AD) (Fig 1).
- There is an extensive body of literature exploring the potential mechanisms of Alzheimer pathology [30–32] with a number of metabolomic studies having examined associations with AD [9–11,33], however few have studied these differences in human brain tissue [15,16]. Our current report identified metabolite compositional differences between three diagnostic groups, i.e., healthy controls, AD, and ASYMADs, i.e., individuals with significant levels of Alzheimer pathology, but with no cognitive impairment during life (S1 Fig). The overall pattern of metabolite shifts observed in the brain was mirrored in each of the individual brain regions (S2 Fig), with the metabolism of PUFAs discriminating between the clinical groups in all brain regions.
- Whilst the CB was included in this study as a brain region showing low levels of Alzheimer pathology, we did observe significant alterations in the levels of the six UFAs in this region relative to controls, suggesting that both Alzheimer pathology and other non-AD-related processes may contribute to or result from the metabolic differences observed in this brain region [34].

Previous studies have examined the abundance of UFAs in the brain of AD patients. Nasaruddin et al. [35] reported that the abundance of 20 fatty acids was increased in Brodmann' 7 region of late stage AD patients. Four of these species, i.e., oleic, linoleic, linolenic, and arachidonic acid were shown to be reduced in our present study, whereas levels of DHA were consistent. Cunnane et al. [36] measured fatty acids in both plasma and brain tissue samples and reported lower levels of esterified DHA in the AD group—specifically in phosphatidylserine in the middle frontal and superior temporal cortices. It must be noted that DHA containing phospholipids are structurally and functionally distinct metabolites to the free fatty acids reported in this study.

The PUFAs can broadly be split into two classes, the omega-3 and omega-6 fatty acids. Alpha-linolenic acid (omega-3) and linoleic acid (omega-6) are both “parental” essential fatty acids, both of which are converted into long chain UFAs, arachidonic acid from linoleic acid and DHA and EPA from linolenic acid. Amtul et al. [37] performed an in vitro study to determine the effect of the omega-6 fatty acids linoleic acid and arachidonic acid, and the omega-9 fatty acid oleic acid on the pathology of AD. Linoleic, arachidonic, and oleic acid were all shown to induce the polymerisation of both tau and Aβ [37], with arachidonic acid also shown to induce Aβ_42_ formation reducing the observed ratio of Aβ_40_/Aβ_42_ [37]. Whilst this information and additional literature [38] would point to oleic acid being pathogenic, there is also a significant body of literature supporting the idea that oleic acid is protective against AD [39,40]. Amtul et al. demonstrated that in in vitro models, oleic acid supplementation reduced secreted Aβ levels and validated these findings in a transgenic mouse model fed an oleic acid-rich diet [39]. The potential benefits of oleic acid supplementation in humans are highlighted by olive oil, which is rich in oleic acid and thought to be protective against age-related cognitive decline and onset of AD [39,41–43], In a clinical trial, Martinez-Lapiscina et al. [44] showed that patients randomised to an olive oil-rich diet had better cognitive function compared to those on a control diet [44].

**Fig 3:**
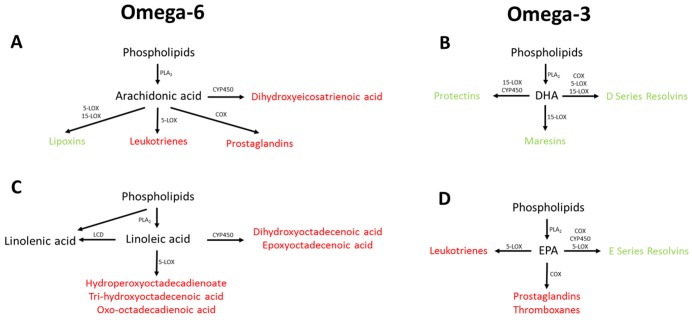
Overview of omega-3 and omega-6 UFA metabolism showing the potential association between the measured UFAs. Metabolites highlighted in red have been associated with increased risk of disease pathology, with metabolites highlighted in green have been associated with decreased risk of disease pathology. COX, cyclooxygenase; CYP450, cytochrome P450; DHA, docosahexaenoic acid; EPA, eicosapentaenoic acid; LCD, lineoyl-CoA desaturase; LOX, lipoxygenase; PLA_2_, phospholipase A_2_.

Dietary supplementation with the omega-3 fatty acids DHA and EPA has also been shown to improve cognitive performance in several animal studies of AD [45–48]. Previous human studies have also reported promising effects on cognition in individuals receiving either DHA, EPA, or a combination of the two [49–51]. The mechanisms by which DHA supplementation may impact AD may be by modulating a beta deposition in the brain [52] 1. The potentially protective effects of EPA in the brain may be mediated through competitive inhibition of the action of lipoxygenases (LOX) (Fig 3), cyclooxygenases (COX), and cytochrome P450 (CYP450) against its omega-6 homologue arachidonic acid (ARA) [53–56]. The breakdown of ARA by COX and 5-LOX produces prostaglandin E_2_ and leukotriene B_4_ [53], which, respectively, are both highly proinflammatory [54], whilst the breakdown of EPA by these enzymes to produce prostaglandin E_3_ and leukotriene B_5_ is less efficient [53] and whilst they are also proinflammatory, they are less potent than the products of ARA [55,56].

UFAs are also precursors of the eicosanoids, which are a large class of highly potent regulatory lipid hormones (Fig 3), possessing a wide range of biological functions. Studies have shown that DHA and EPA containing phosphatidylcholines (PCs) are reduced in abundance in the blood of patients with AD [12,57,58]. It has been reported that COX, LOX, and CYP450, i.e., enzymes that break down EPA and the other five UFAs measured, have all been shown to be up-regulated in patients with AD [59,60], potentially explaining the decrease in the observed UFA species. Breakdown of EPA and other UFAs by COX leads to the production of prostaglandins with LOX breakdown leading to the production of leukotrienes, and breakdown by CYP450 producing epoxyeicosatrienoic acid (Fig 3), all of which have been associated with pathology in a range of diseases including AD [61–63]. The breakdown of DHA by these three enzymes produces resolvins, maresins, and protectins, which have been shown to be protective against numerous disease pathologies [64–66], including in AD [67,68].

### Strengths and limitations

The main strengths of this study are the well-characterized longitudinal BLSA cohort with serial cognitive assessments and detailed neuropathological examination at death. Inclusion of the “ASYMAD” group of individuals allows us to relate the observed shifts in metabolism with both severity of pathology and the expression of AD symptoms. Inclusion of distinct brain regions representing areas both vulnerable and resistant to AD pathology is another key strength of this study

We applied four distinct metabolomic assays utilising a range of complimentary instruments and chromatographic techniques that together provided a wide coverage of the brain metabolome.

The main limitation of this study is the relatively small sample size There are few cohorts with extensive longitudinal cognitive assessments during life and detailed neuropathological assessments at death as the BLSA. Our findings therefore merit confirmation in larger studies. Another limitation of this study is one shared by all nontargeted metabolomics approaches, which is the difficulty in assigning metabolite identities to metabolite features within the dataset. With the advent of large publicly available metabolite databases, it is hoped that larger numbers of metabolites will be identified in future studies using these methods.

In summary, we identified significant differences in the abundance of six UFAs in three brain regions with gradations in these metabolites being related to both severity of neuropathology at death as well as domain-specific cognitive performance during life. Our work suggests that dysregulation of UFA’s metabolism plays a role in driving AD pathology and that these results provide further evidence for the metabolic basis of AD pathogenesis.

## Supporting information

S1 STROBE Checklist (DOC)

S1 Table Summary of the association of all annotated metabolites with disease pathology. ^a^ *p*-value calculated using mann-whitney U-test. ^b^ fold change relative to controls. ^c^ fold change relative to asymptomatic. AMP; adenosine-monophosphate, Asymp; asymptomatic, Cont; control, Dem; dementia/Alzheimer, GABA; gamma-aminobutanoate, L-DOPA; L-dihydroxy-phenylalanine.
(DOCX)

S2 Table Summary of the association of all annotated metabolites with brain region. ^a^ *p*-value calculated using mann-whitney U-test. ^b^ fold change relative to CB, ^c^ fold change relative to ITG. AMP; adenosine-monophosphate, CB; cerebellum, GABA; gamma-aminobutanoate, ITG; inferior temporal gyrus, L-DOPA; L-dihydroxy-phenylalanine, MFG; medial frontal gyrus.
(DOCX)

S3 Table Correlation of the abundance of six UFAs with measures of both cross sectional and longitudinal memory performance. Relationships between global measures of cross sectional and longitudinal memory performance and the regional abundances of six UFAs, values highlighted in bold are significant at *p* < 0.05. ^*^ correlation of fatty acid abundance to last memory score before death, ^+^ correlation of fatty acid abundance to rate of longitudinal decline in memory. CERAD; Consortium to Establish a Registry for Alzheimer’s Disease.
(DOCX)

S4 Table Correlation of the abundance of six UFAs with measures of both cross sectional and longitudinal language performance. Relationships between global measures of cross sectional and longitudinal language performance and the regional abundances of six UFAs, values highlighted in bold are significant at *p* < 0.05. ^*^ correlation of fatty acid abundance to last language score before death, ^+^ correlation of fatty acid abundance to rate of longitudinal decline in language. CERAD; Consortium to Establish a Registry for Alzheimer’s Disease.
(DOCX)

S5 Table Correlation of the abundance of six UFAs with measures of both cross sectional and longitudinal attention span. Relationships between global measures of cross sectional and longitudinal attention span performance and the regional abundances of six UFAs, values highlighted in bold are significant at *p* < 0.05. ^*^ correlation of fatty acid abundance to last attention span score before death, ^+^ correlation of fatty acid abundance to rate of longitudinal decline in attention span. CERAD; Consortium to Establish a Registry for Alzheimer’s Disease.
(DOCX)

S6 Table Correlation of the abundance of six UFAs with measures of both cross sectional and longitudinal executive function. Relationships between global measures of cross sectional and longitudinal attention span performance and the regional abundances of six UFAs, values highlighted in bold are significant at *p* < 0.05. ^*^ correlation of fatty acid abundance to last executive function score before death, ^+^ correlation of fatty acid abundance to rate of longitudinal decline in executive function score. CERAD; Consortium to Establish a Registry for Alzheimer’s Disease.
(DOCX)

S7 Table Correlation of the abundance of six UFAs with measures of both cross sectional and longitudinal visual spatial awareness. Relationships between global measures of cross sectional and longitudinal attention span performance and the regional abundances of six UFAs, values highlighted in bold are significant at *p* < 0.05. ^*^ correlation of fatty acid abundance to last visual spatial awareness score before death, ^+^ correlation of fatty acid abundance to rate of longitudinal decline in visual spatial awareness score. CERAD; Consortium to Establish a Registry for Alzheimer’s Disease.
(DOCX)

S1 Fig Scores plots from curated PLS-DA models comparing the metabolite composition of brain regions and diagnostic groups. A) Comparison of metabolite composition of CB, ITG, and MFG (R^2^X = 0.639, R^2^Y = 0.622, Q^2^ = 0.608, CV-ANOVA = 5.65 × 10^−33^) B) Comparison of metabolite composition of diagnostic groups, control, asymptomatic, and individuals with dementia (R^2^X = 0.499, R^2^Y = 0.439, Q^2^ = 0.404, CV-ANOVA = 4.72 × 10^−21^).
(TIF)

S2 Fig Scores plots from curated PLS-DA models comparing the metabolite composition of diagnostic groups in individual brain regions. A) Comparison of metabolite composition of diagnostic groups, control, asymptomatic, and individuals with dementia in CB samples (R^2^X = 0.527, R^2^Y = 0.539, Q^2^ = 0.424, CV-ANOVA = 5.04 × 10^−4^), B) Comparison of metabolite composition of diagnostic groups, control, asymptomatic, and individuals with dementia in ITG samples (R^2^X = 0.592, R^2^Y = 0.563, Q^2^ = 0.424, CV-ANOVA = 3.28 × 10^−7^), C) Comparison of metabolite composition of diagnostic groups, control, asymptomatic, and individual with dementia in MFG samples (R^2^X = 0.650, R^2^Y = 0.563, Q^2^ = 0.490, CV-ANOVA = 1.62 × 10^−5^).
(TIF)

S3 Fig Forest plot showing the relationship of the abundance of six UFAs in the CB and five measures of cognition. **A)** association between fatty acid abundance of UFA levels and longitudinal cognitive performance **B)** associations between fatty acid abundance cognitive performance at the last visit prior to death. AA: arachidonic acid, DHA: docosahexaenoic acid, EPA: eicosapentaenoic acid, LA: linoleic acid, LNA: linolenic acid, OLA: oleic acid.
(TIF)

S4 Fig Forest plot showing the relationship of the abundance of six UFAs in the ITG and five measures of cognition. **A)** association between fatty acid abundance of UFA levels and longitudinal cognitive performance **B)** associations between fatty acid abundance cognitive performance at the last visit prior to death. AA: arachidonic acid, DHA: docosahexaenoic acid, EPA: eicosapentaenoic acid, LA: linoleic acid, LNA: linolenic acid, OLA: oleic acid.
(TIF)

S1 Text Analysis plan. (DOCX)

## Abbreviations

Aβ: amyloid-β
AD: Alzheimer disease
ASYMAD: asymptomatic Alzheimer’s disease
BLSA: Baltimore Longitudinal Study of Aging
CB: cerebellum; CERAD, Consortium to Establish a Registry for Alzheimer’s Disease
COX: cyclooxygenase
CYP450: cytochrome P450
DHA: docosahexaenoic acid
EPA: eicosapentaenoic acid
GC-MS: gas chromatography–mass spectrometry
ITG: inferior temporal gyrus; LC-MS, liquid chromatography–mass spectrometry
LOX: lipoxygenase
MFG: middle frontal gyrus
PLS-DA: partial least square–discriminant analysis
PUFA: poly-unsaturated fatty acid
UFA: unsaturated fatty acid
