# Supplementary material for: Association between fatty acid metabolism in the brain and Alzheimer disease neuropathology and cognitive performance: A nontargeted metabolomic study
Source: PLoS Med. 2017 Mar 21;14(3):e1002266. doi: 10.1371/journal.pmed.1002266 (PMC5360226; doi:10.1371/journal.pmed.1002266)
Supplement: S1 STROBE Checklist — (DOC) [file pmed.1002266.s001.doc]

STROBE Statement—checklist of items that should be included in reports of observational studies

|  | Item No | Recommendation | Page No. | Relevant text from manuscript |
| --- | --- | --- | --- | --- |
| **Title and abstract** | 1 | (*a*) Indicate the study’s design with a commonly used term in the title or the abstract | 1 | “…*a non-targeted metabolomics study*.” |
|  |  | (*b*) Provide in the abstract an informative and balanced summary of what was done and what was found | 2 | See Section: Methods and Findings |
| Introduction | | |  |  |
| Background/rationale | 2 | Explain the scientific background and rationale for the investigation being reported | **Introduction** Paragraph 3 | “*In AD, there is a regional specificity in the vulnerability*…” |
| Objectives | 3 | State specific objectives, including any pre-specified hypotheses |  |  |
| Methods | | |  |  |
| Study design | 4 | Present key elements of study design early in the paper | **Methods and Materials**  Paragraph 1,2,3,4,5,6,7,8 | See ‘*Materials and Methods*’  ‘*Sample Information*’  ‘*Chemicals and Reagents*’  ‘*Sample Preparation*’  ‘*Metabolite Acquisition*’  ‘*Data Processing*’ |
| Setting | 5 | Describe the setting, locations, and relevant dates, including periods of recruitment, exposure, follow-up, and data collection | **Materials and Methods**  Paragraph 1,2 | “*The BLSA is a prospective, ongoing cohort study of community-dwelling volunteer participants in Baltimore beginning in 1958. As such, it is among the largest and longest-running longitudinal studies of aging in the United States (22, 23). In general, at the time of entry into the study, participants had no physical or cognitive impairment. Detailed examinations, including neuropsychological assessments and neurological, laboratory, and radiological evaluations, were conducted every 2 years. Since 2003, participants older than 80 years have received yearly assessments.*” |
| Participants | 6 | (*a*) *Cohort study*—Give the eligibility criteria, and the sources and methods of selection of participants. Describe methods of follow-up  *Case-control study*—Give the eligibility criteria, and the sources and methods of case ascertainment and control selection. Give the rationale for the choice of cases and controls  *Cross-sectional study*—Give the eligibility criteria, and the sources and methods of selection of participants | **Materials and Methods**  Paragraph 1,2 | “*The BLSA is a prospective, ongoing cohort study of community-dwelling volunteer participants in Baltimore beginning in 1958. As such, it is among the largest and longest-running longitudinal studies of aging in the United States (22, 23). In general, at the time of entry into the study, participants had no physical or cognitive impairment. Detailed examinations, including neuropsychological assessments and neurological, laboratory, and radiological evaluations, were conducted every 2 years. Since 2003, participants older than 80 years have received yearly assessments.*” |
|  |  | (*b*) *Cohort study*—For matched studies, give matching criteria and number of exposed and unexposed  *Case-control study*—For matched studies, give matching criteria and the number of controls per case | **N/A** | **N/A** |
| Variables | 7 | Clearly define all outcomes, exposures, predictors, potential confounders, and effect modifiers. Give diagnostic criteria, if applicable | **Methods and Materials**  Paragraph 1,2,3,4,5,6,7,8 | See ‘*Materials and Methods*’  ‘*Sample Information*’  ‘*Chemicals and Reagents*’  ‘*Sample Preparation*’  ‘*Metabolite Acquisition*’  ‘*Data Processing*’ |
| Data sources/ measurement | 8* | For each variable of interest, give sources of data and details of methods of assessment (measurement). Describe comparability of assessment methods if there is more than one group | **Methods and Materials**  Paragraph 1,2,3,4,5,6,7,8 | See ‘*Materials and Methods*’  ‘*Sample Information*’  ‘*Chemicals and Reagents*’  ‘*Sample Preparation*’  ‘*Metabolite Acquisition*’  ‘*Data Processing*’ |
| Bias | 9 | Describe any efforts to address potential sources of bias | **Methods and Materials**  Paragraph 2 | “*As reported previously, the autopsy subsample is not significantly different from the BLSA cohort as a whole in terms of the rates of dementia and clinical stroke (28)*.” |
| Study size | 10 | Explain how the study size was arrived at |  |  |
| Quantitative variables | 11 | Explain how quantitative variables were handled in the analyses. If applicable, describe which groupings were chosen and why | **Materials and Methods** Paragraph 6,7,8 | See section: ‘*Data Processing*’ |
| Statistical methods | 12 | (*a*) Describe all statistical methods, including those used to control for confounding | **Materials and Methods** Paragraph 6,7,8 | See section: ‘*Data Processing*’ |
|  |  | (*b*) Describe any methods used to examine subgroups and interactions | **N/A** | **N/A** |
|  |  | (*c*) Explain how missing data were addressed | **N/A** | **N/A** |
|  |  | (*d*) *Cohort study*—If applicable, explain how loss to follow-up was addressed  *Case-control study*—If applicable, explain how matching of cases and controls was addressed  *Cross-sectional study*—If applicable, describe analytical methods taking account of sampling strategy | **N/A** | **N/A** |
|  |  | (*e*) Describe any sensitivity analyses |  |  |

| Results | | |  |  |
| --- | --- | --- | --- | --- |
| Participants | 13* | (a) Report numbers of individuals at each stage of study—eg numbers potentially eligible, examined for eligibility, confirmed eligible, included in the study, completing follow-up, and analysed | **N/A** | See information in Table 1 |
|  |  | (b) Give reasons for non-participation at each stage | **N/A** | **N/A** |
|  |  | (c) Consider use of a flow diagram | **N/A** | **N/A** |
| Descriptive data | 14* | (a) Give characteristics of study participants (eg demographic, clinical, social) and information on exposures and potential confounders | **Methods and Materials** Paragraph 2 | “*Table 1 describes the demographic characteristics of the participants whose brain tissue samples were used in this study*.” |
|  |  | (b) Indicate number of participants with missing data for each variable of interest | **N/A** | There were no participants in this study which had any missing data. |
|  |  | (c) *Cohort study*—Summarise follow-up time (eg, average and total amount) | **Methods and Materials** Paragraph 2 | “*Briefly, the mean age at death in the autopsy sample is 88.3 ± 7.3 years (range 69.3–103.2), and the mean interval between last evaluation and death is 8.7± 6.7 months (27)*.” |
| Outcome data | 15* | *Cohort study*—Report numbers of outcome events or summary measures over time | **N/A** | **N/A** |
|  |  | *Case-control study—*Report numbers in each exposure category, or summary measures of exposure | **N/A** | **N/A** |
|  |  | *Cross-sectional study—*Report numbers of outcome events or summary measures | **N/A** | **N/A** |
| Main results | 16 | (*a*) Give unadjusted estimates and, if applicable, confounder-adjusted estimates and their precision (eg, 95% confidence interval). Make clear which confounders were adjusted for and why they were included | **Results** Paragraph 2 | Owing to the small sample size no correction was performed on this data “*Multivariate models were constructed based on the uncorrected abundance of all 4,897 metabolite features to assess the effects of both brain region and pathological diagnosis on metabolite composition.*” |
|  |  | (*b*) Report category boundaries when continuous variables were categorized | **N/A** | **N/A** |
|  |  | (*c*) If relevant, consider translating estimates of relative risk into absolute risk for a meaningful time period | **N/A** | **N/A** |
| Other analyses | 17 | Report other analyses done—eg analyses of subgroups and interactions, and sensitivity analyses | **Results** Paragraph 4,5 | “*Table 3 and Fig.1 summarize results of analyses comparing brain tissue UFA levels between the three diagnostic groups in each of three brain regions*...”  “*As well as associating with disease status, levels of all six unsaturated fatty acids in the MFG and ITG were shown to correlate significantly with both measures of neurofibrillary pathology estimated by Braak score and amyloid plaque burden assessed by the CERAD score (Table 4).*”  “*Assessments of brain tissue levels of these UFAs in relation to domain-specific measures of cognitive performance showed consistent patterns across domains and several significant cross sectional and longitudinal associations across the three brain regions were found (S3-7 Table and S3 and S4 Fig)*…” |
| Discussion | | |  |  |
| Key results | 18 | Summarise key results with reference to study objectives | **Discussion** Paragraph 9 | “…*we identified significant changes in the abundance of six UFAs in three brain regions with gradations in these metabolites being related to both severity of neuropathology at death as well as domain-specific cognitive performance during life.*” |
| Limitations | 19 | Discuss limitations of the study, taking into account sources of potential bias or imprecision. Discuss both direction and magnitude of any potential bias | **Discussion** Paragraph 6,7,8 | See ‘*Strengths and Limitations*’ section |
| Interpretation | 20 | Give a cautious overall interpretation of results considering objectives, limitations, multiplicity of analyses, results from similar studies, and other relevant evidence | **Discussion** Paragraph 9 | “*Our work suggests that dysregulation of UFA’s metabolism plays a role in driving AD pathology*…” |
| Generalisability | 21 | Discuss the generalisability (external validity) of the study results | **N/A** | **N/A** |
| Other information | | |  |  |
| Funding | 22 | Give the source of funding and the role of the funders for the present study and, if applicable, for the original study on which the present article is based | **Acknowledgements** | “*This work has been supported by grants from the Libyan Cultural attaché of Libyan embassy and the European Medical Information Framework – Alzheimer’s Disease (EMIF-AD)*.” |

*Give information separately for cases and controls in case-control studies and, if applicable, for exposed and unexposed groups in cohort and cross-sectional studies.

**Note:** An Explanation and Elaboration article discusses each checklist item and gives methodological background and published examples of transparent reporting. The STROBE checklist is best used in conjunction with this article (freely available on the Web sites of PLoS Medicine at http://www.plosmedicine.org/, Annals of Internal Medicine at http://www.annals.org/, and Epidemiology at http://www.epidem.com/). Information on the STROBE Initiative is available at www.strobe-statement.org.
