# Supplementary material for: Association between fatty acid metabolism in the brain and Alzheimer disease neuropathology and cognitive performance: A nontargeted metabolomic study
Source: PLoS Med. 2017 Mar 21;14(3):e1002266. doi: 10.1371/journal.pmed.1002266 (PMC5360226; doi:10.1371/journal.pmed.1002266)
Supplement: S1 Table — a p-value calculated using mann-whitney U-test. b fold change relative to controls. c fold change relative to asymptomatic. AMP; adenosine-monophosphate, Asymp; asymptomatic, Cont; control, Dem; dementia/Alzheimer, GABA; gamma-aminobutanoate, L-DOPA; L-dihydroxy-phenylalanine. (DOCX) [file pmed.1002266.s002.docx]

**S1 Table Summary of the association of all annotated metabolites with disease pathology.**

|  | **Cont Vs Asymp** | | **Cont Vs Dem** | | **Asymp Vs Dem** | |
| --- | --- | --- | --- | --- | --- | --- |
|  | **p-value^a^** | **FC^b^** | **p-value^a^** | **FC^b^** | **p-value^a^** | **FC^c^** |
| Acetylaspartate | 4.7×10^-2^ | 0.93 | 9.1×10^-3^ | 0.91 | 6.5×10^-1^ | 0.98 |
| Acetylaspartylglutamate | 8.4×10^-1^ | 0.99 | 9.1×10^-2^ | 0.90 | 1.5×10^-1^ | 0.91 |
| Acetylcarnitine | 5.0×10^-2^ | 1.21 | 1.1×10^-2^ | 1.28 | 5.5×10^-1^ | 1.06 |
| Adenine | 9.2×10^-2^ | 1.10 | 8.0×10^-4^ | 1.18 | 1.4×10^-1^ | 1.08 |
| Adenosine | 1.2×10^-1^ | 1.23 | 9.5×10^-1^ | 0.99 | 1.0×10^-1^ | 0.81 |
| Adenosylmethionine | 9.1×10^-1^ | 0.98 | 4.4×10^-3^ | 0.68 | 2.1×10^-3^ | 0.69 |
| Alanine | 7.4×10^-1^ | 1.02 | 1.6×10^-2^ | 0.87 | 2.5×10^-2^ | 0.85 |
| Allantoin | 7.0×10^-3^ | 1.19 | 4.7×10^-4^ | 1.30 | 2.0×10^-1^ | 1.09 |
| Aminobutanal | 6.8×10^-2^ | 0.94 | 9.2×10^-5^ | 0.86 | 6.9×10^-2^ | 0.92 |
| AMP | 6.0×10^-1^ | 0.90 | 3.3×10^-2^ | 1.50 | 9.4×10^-3^ | 1.66 |
| Arachidonic acid | 9.5×10^-2^ | 0.88 | 2.9×10^-5^ | 0.75 | 1.4×10^-2^ | 0.84 |
| Arginine | 5.7×10^-2^ | 1.24 | 1.1×10^-3^ | 1.38 | 2.9×10^-1^ | 1.12 |
| Ascorbate | 1.6×10^-1^ | 1.20 | 1.9×10^-5^ | 1.54 | 6.2×10^-3^ | 1.29 |
| Asparagine | 2.4×10^-1^ | 1.17 | 3.0×10^-2^ | 1.29 | 4.3×10^-1^ | 1.10 |
| Aspartate | 6.3×10^-2^ | 0.91 | 1.5×10^-4^ | 0.84 | 1.0×10^-1^ | 0.92 |
| Benzesulfonamide | 6.7×10^-2^ | 0.76 | 4.6×10^-7^ | 0.40 | 6.7×10^-4^ | 0.52 |
| Butyrylcarnitine | 1.9×10^-1^ | 1.17 | 1.2×10^-2^ | 1.40 | 1.4×10^-1^ | 1.19 |
| Carbamic acid | 1.9×10^-1^ | 1.15 | 2.0×10^-7^ | 1.53 | 4.0×10^-4^ | 1.32 |
| Carnitine | 7.5×10^-1^ | 0.98 | 7.2×10^-1^ | 1.02 | 5.6×10^-1^ | 1.04 |
| Carnosine | 2.9×10^-1^ | 0.91 | 2.0×10^-1^ | 1.21 | 6.6×10^-2^ | 1.33 |
| Cholestenol | 1.1×10^-1^ | 1.17 | 1.0×10^-7^ | 1.44 | 3.0×10^-3^ | 1.24 |
| Cholesterol | 7.6×10^-3^ | 0.74 | 6.9×10^-8^ | 0.53 | 1.1×10^-2^ | 0.72 |
| Citrulline | 1.7×10^-1^ | 1.25 | 7.4×10^-1^ | 0.96 | 1.2×10^-1^ | 0.77 |
| Coumaric acid | 9.8×10^-2^ | 1.16 | 1.1×10^-3^ | 1.28 | 2.3×10^-1^ | 1.10 |
| Creatine | 1.6×10^-1^ | 0.95 | 3.2×10^-2^ | 0.92 | 4.5×10^-1^ | 0.97 |
| Creatinine | 5.1×10^-1^ | 0.98 | 3.5×10^-1^ | 0.98 | 8.7×10^-1^ | 1.00 |
| Csytathionine | 6.3×10^-1^ | 1.09 | 7.3×10^-1^ | 1.06 | 9.0×10^-1^ | 0.98 |
| Cysteine | 4.7×10^-1^ | 1.06 | 1.1×10^-4^ | 1.33 | 2.1×10^-3^ | 1.26 |
| Cytidine | 2.8×10^-1^ | 1.14 | 8.5×10^-3^ | 1.28 | 2.2×10^-1^ | 1.13 |
| Cytosine | 8.4×10^-1^ | 0.98 | 7.4×10^-1^ | 1.03 | 6.4×10^-1^ | 1.06 |
| Dehydroascorbic acid | 8.6×10^-1^ | 0.94 | 1.9×10^-2^ | 2.14 | 1.4×10^-2^ | 2.28 |
| Deoxyflurouridine | 5.3×10^-1^ | 1.08 | 1.0×10^-3^ | 1.45 | 2.1×10^-2^ | 1.34 |
| Deoxy-Ribose | 6.1×10^-1^ | 0.94 | 3.6×10^-1^ | 0.90 | 6.7×10^-1^ | 0.95 |
| Dimethylglycine | 2.9×10^-2^ | 0.89 | 1.1×10^-6^ | 0.74 | 2.3×10^-4^ | 0.84 |
| Docosahexanoic acid | 1.8×10^-1^ | 1.14 | 1.7×10^-7^ | 1.45 | 1.6×10^-3^ | 1.27 |
| Dopamine | 1.4×10^-1^ | 0.91 | 4.7×10^-3^ | 0.82 | 2.3×10^-1^ | 0.90 |
| Eicosapentaenoic acid | 1.6×10^-3^ | 0.25 | 4.4×10^-4^ | 0.16 | 1.8×10^-1^ | 0.63 |
| Fumaric acid | 4.5×10^-2^ | 0.91 | 1.9×10^-4^ | 0.85 | 1.6×10^-1^ | 0.93 |
| GABA | 3.6×10^-1^ | 1.07 | 4.4×10^-3^ | 1.18 | 8.7×10^-2^ | 1.11 |
| Galactofuranose | 5.7×10^-1^ | 1.06 | 6.8×10^-2^ | 1.17 | 2.3×10^-1^ | 1.11 |
| Gluconic acid | 1.5×10^-2^ | 1.31 | 1.0×10^-4^ | 1.51 | 1.6×10^-1^ | 1.15 |
| Glucose | 5.1×10^-1^ | 0.95 | 3.3×10^-1^ | 1.07 | 1.4×10^-1^ | 1.13 |
| Glutamate | 9.5×10^-1^ | 1.00 | 1.0×10^-1^ | 0.96 | 1.9×10^-1^ | 0.96 |
| Glutamine | 7.1×10^-1^ | 1.02 | 8.3×10^-1^ | 0.99 | 5.4×10^-1^ | 0.97 |
| Glutathione | 8.3×10^-1^ | 1.02 | 1.3×10^-1^ | 1.13 | 3.4×10^-1^ | 1.10 |
| Glycine | 3.8×10^-1^ | 1.07 | 2.6×10^-1^ | 0.93 | 8.8×10^-2^ | 0.87 |
| Guanidobutanoate | 2.0×10^-1^ | 0.94 | 3.7×10^-6^ | 0.75 | 5.9×10^-5^ | 0.80 |
| Guanine | 4.5×10^-2^ | 0.83 | 1.0×10^-3^ | 0.75 | 3.2×10^-1^ | 0.91 |
| Guanosine | 7.5×10^-2^ | 0.83 | 5.0×10^-3^ | 0.76 | 4.4×10^-1^ | 0.92 |
| Hexanedioic acid | 2.8×10^-1^ | 1.11 | 6.4×10^-7^ | 1.42 | 5.3×10^-4^ | 1.28 |
| Histidine | 9.1×10^-1^ | 0.99 | 7.6×10^-3^ | 0.71 | 7.8×10^-3^ | 0.72 |
| Homocysteine | 8.0×10^-1^ | 0.97 | 2.0×10^-1^ | 1.14 | 1.6×10^-1^ | 1.17 |
| Hydroxyanthranillate | 5.4×10^-1^ | 0.95 | 3.6×10^-1^ | 0.92 | 7.4×10^-1^ | 0.97 |
| Hydroxyguanine | 8.3×10^-2^ | 3.06 | 2.1×10^-1^ | 1.05 | 9.0×10^-2^ | 0.34 |
| Hypoxanthine | 4.7×10^-1^ | 0.98 | 5.5×10^-4^ | 0.88 | 4.1×10^-3^ | 0.91 |
| Indoleacetic acid | 1.4×10^-1^ | 0.93 | 2.6×10^-4^ | 0.82 | 4.8×10^-2^ | 0.89 |
| Inosine | 2.8×10^-1^ | 0.93 | 8.9×10^-1^ | 0.99 | 3.1×10^-1^ | 1.07 |
| Kynurenic acid | 7.3×10^-1^ | 0.96 | 3.2×10^-1^ | 0.87 | 5.2×10^-1^ | 0.91 |
| Lactic acid | 9.1×10^-1^ | 1.00 | 7.1×10^-1^ | 0.99 | 8.2×10^-1^ | 0.99 |
| L-DOPA | 9.3×10^-2^ | 1.11 | 1.9×10^-4^ | 1.23 | 6.1×10^-2^ | 1.10 |
| Leucine/Isoleucine | 4.5×10^-1^ | 1.05 | 3.4×10^-2^ | 1.14 | 1.7×10^-1^ | 1.09 |
| Linoleic acid | 7.0×10^-3^ | 0.74 | 8.9×10^-8^ | 0.52 | 6.7×10^-3^ | 0.71 |
| Linolenic acid | 7.5×10^-2^ | 0.90 | 2.6×10^-4^ | 0.84 | 1.6×10^-1^ | 0.93 |
| Lysine | 1.9×10^-1^ | 1.20 | 3.1×10^-2^ | 1.41 | 2.9×10^-1^ | 1.18 |
| Malate | 9.8×10^-1^ | 1.00 | 1.8×10^-1^ | 0.91 | 2.7×10^-1^ | 0.91 |
| Methionine | 4.2×10^-1^ | 1.09 | 2.1×10^-1^ | 1.14 | 6.1×10^-1^ | 1.05 |
| Methyl-aspartic acid | 8.2×10^-1^ | 0.99 | 4.6×10^-2^ | 0.88 | 7.5×10^-2^ | 0.89 |
| Methylheptadecadiynoate | 2.5×10^-2^ | 0.79 | 2.8×10^-7^ | 0.59 | 1.2×10^-2^ | 0.75 |
| Methylstearate | 5.4×10^-1^ | 0.85 | 2.6×10^-2^ | 0.56 | 2.0×10^-1^ | 0.66 |
| Nicotinamide | 6.9×10^-1^ | 0.98 | 6.8×10^-1^ | 0.98 | 9.6×10^-1^ | 1.00 |
| Nicotinic acid | 2.5×10^-1^ | 1.13 | 1.7×10^-2^ | 0.80 | 1.7×10^-3^ | 0.71 |
| Nitrotyrosine | 9.6×10^-3^ | 1.25 | 1.6×10^-3^ | 1.33 | 4.9×10^-1^ | 1.06 |
| Noradrenaline | 2.9×10^-1^ | 1.16 | 9.8×10^-3^ | 1.35 | 1.9×10^-1^ | 1.16 |
| Octadecanal | 1.6×10^-1^ | 2.41 | 5.6×10^-1^ | 0.68 | 5.5×10^-2^ | 0.28 |
| Oleic acid | 5.5×10^-2^ | 0.73 | 3.3×10^-7^ | 0.34 | 3.9×10^-4^ | 0.46 |
| Ornithine | 8.5×10^-2^ | 1.18 | 2.1×10^-3^ | 1.29 | 3.1×10^-1^ | 1.09 |
| Oxoarginine | 9.0×10^-1^ | 0.99 | 8.6×10^-4^ | 0.77 | 2.3×10^-2^ | 0.78 |
| Oxoglutarate | 9.3×10^-1^ | 0.99 | 5.5×10^-2^ | 1.10 | 1.1×10^-1^ | 1.11 |
| Palmitic acid | 2.0×10^-2^ | 0.73 | 4.9×10^-7^ | 0.44 | 1.9×10^-3^ | 0.61 |
| Pantothenate | 3.3×10^-1^ | 0.88 | 3.3×10^-2^ | 0.75 | 1.1×10^-1^ | 0.85 |
| Phenylalanine | 2.1×10^-1^ | 1.09 | 5.5×10^-2^ | 1.14 | 4.3×10^-1^ | 1.05 |
| Phosphocholine | 3.5×10^-1^ | 1.06 | 9.5×10^-3^ | 1.14 | 2.6×10^-1^ | 1.07 |
| Phosphocreatine | 3.7×10^-1^ | 0.87 | 1.1×10^-1^ | 0.77 | 4.4×10^-1^ | 0.88 |
| Proline | 2.6×10^-1^ | 1.06 | 8.5×10^-1^ | 1.01 | 3.4×10^-1^ | 0.95 |
| Propionylcarnitine | 1.3×10^-1^ | 1.29 | 2.7×10^-2^ | 1.37 | 6.5×10^-1^ | 1.07 |
| Serine | 6.9×10^-1^ | 1.04 | 6.7×10^-1^ | 0.96 | 4.3×10^-1^ | 0.92 |
| Spermidine | 8.4×10^-1^ | 0.95 | 1.8×10^-1^ | 0.72 | 2.1×10^-1^ | 0.76 |
| Succinate | 6.6×10^-1^ | 0.96 | 7.0×10^-2^ | 1.13 | 3.4×10^-2^ | 1.18 |
| Taurine | 5.0×10^-1^ | 0.96 | 9.3×10^-1^ | 1.01 | 4.7×10^-1^ | 1.04 |
| Threonine | 3.4×10^-1^ | 1.07 | 8.9×10^-2^ | 1.11 | 5.6×10^-1^ | 1.04 |
| Tryptophan | 1.5×10^-1^ | 1.16 | 1.2×10^-1^ | 1.19 | 7.8×10^-1^ | 1.03 |
| Tyrosine | 2.6×10^-2^ | 1.22 | 2.7×10^-3^ | 1.30 | 4.4×10^-1^ | 1.07 |
| Uracil | 8.5×10^-1^ | 1.01 | 1.8×10^-3^ | 1.22 | 1.8×10^-3^ | 1.21 |
| Uric acid | 5.9×10^-1^ | 1.06 | 4.5×10^-2^ | 0.80 | 2.0×10^-2^ | 0.76 |
| Uridine | 9.6×10^-1^ | 1.00 | 1.3×10^-1^ | 0.88 | 1.5×10^-1^ | 0.89 |
| Valine | 4.1×10^-2^ | 1.21 | 5.6×10^-3^ | 1.25 | 7.6×10^-1^ | 1.03 |
| Xanthine | 3.2×10^-1^ | 1.04 | 1.2×10^-1^ | 0.95 | 2.0×10^-2^ | 0.92 |
| Xanthosine | 9.7×10^-1^ | 1.00 | 7.5×10^-1^ | 1.02 | 7.3×10^-1^ | 1.03 |

^a^ p-value calculated using mann-whitney U-test. ^b^ fold change relative to controls, ^c^ fold change relative to asymptomatic. AMP; adenosine-monophosphate, Asymp; asymptomatic, Cont; control, Dem; dementia, GABA; gamma-aminobutanoate, L-DOPA; L-dihydroxy-phenylalanine.
