# Supplementary material for: Association between fatty acid metabolism in the brain and Alzheimer disease neuropathology and cognitive performance: A nontargeted metabolomic study
Source: PLoS Med. 2017 Mar 21;14(3):e1002266. doi: 10.1371/journal.pmed.1002266 (PMC5360226; doi:10.1371/journal.pmed.1002266)
Supplement: S2 Table — a p-value calculated using mann-whitney U-test. b fold change relative to CB, c fold change relative to ITG. AMP; adenosine-monophosphate, CB; cerebellum, GABA; gamma-aminobutanoate, ITG; inferior temporal gyrus, L-DOPA; L-dihydroxy-phenylalanine, MFG; medial frontal gyrus. (DOCX) [file pmed.1002266.s003.docx]

**S2 Table Summary of the association of all annotated metabolites with brain region.**

|  | **CB Vs ITG** | | **CB Vs MFG** | | **ITG Vs MFG** | |
| --- | --- | --- | --- | --- | --- | --- |
|  | **p-value^a^** | **FC^b^** | **p-value^a^** | **FC^b^** | **p-value^a^** | **FC^c^** |
| Acetylaspartate | 9.4×10^-3^ | 1.11 | 3.1×10^-3^ | 1.12 | 7.5×10^-1^ | 1.01 |
| Acetylaspartylglutamate | 1.3×10^-13^ | 0.64 | 4.9×10^-6^ | 0.80 | 2.5×10^-5^ | 1.26 |
| Acetylcarnitine | 3.7×10^-5^ | 0.66 | 2.1×10^-5^ | 0.64 | 7.5×10^-1^ | 0.98 |
| Adenine | 4.3×10^-3^ | 0.88 | 6.8×10^-8^ | 0.77 | 6.4×10^-3^ | 0.87 |
| Adenosine | 7.3×10^-1^ | 1.04 | 8.2×10^-4^ | 1.58 | 9.5×10^-4^ | 1.51 |
| Adenosylmethionine | 2.9×10^-3^ | 0.67 | 2.7×10^-1^ | 0.86 | 4.7×10^-2^ | 1.29 |
| Alanine | 1.3×10^-1^ | 0.96 | 6.3×10^-2^ | 0.95 | 8.3×10^-1^ | 0.99 |
| Allantoin | 4.5×10^-1^ | 0.95 | 4.6×10^-4^ | 0.78 | 5.8×10^-3^ | 0.82 |
| Aminobutanal | 2.4×10^-4^ | 1.16 | 5.1×10^-10^ | 1.27 | 1.5×10^-2^ | 1.10 |
| AMP | 7.6×10^-2^ | 0.70 | 9.4×10^-4^ | 0.44 | 3.8×10^-3^ | 0.63 |
| Arachidonic acid | 3.4×10^-1^ | 1.07 | 2.4×10^-2^ | 1.19 | 1.3×10^-1^ | 1.08 |
| Arginine | 1.7×10^-3^ | 0.79 | 1.1×10^-7^ | 0.63 | 1.8×10^-2^ | 0.80 |
| Ascorbate | 1.2×10^-1^ | 1.19 | 4.3×10^-1^ | 1.10 | 4.5×10^-1^ | 0.93 |
| Asparagine | 1.3×10^-2^ | 0.77 | 1.0×10^-7^ | 0.49 | 1.1×10^-4^ | 0.63 |
| Aspartate | 1.8×10^-3^ | 1.16 | 6.8×10^-8^ | 1.29 | 2.8×10^-2^ | 1.11 |
| Benzesulfonamide | 6.1×10^-1^ | 0.91 | 1.6×10^-1^ | 1.26 | 4.9×10^-2^ | 1.39 |
| Butyrylcarnitine | 3.7×10^-5^ | 0.56 | 2.0×10^-3^ | 0.67 | 8.1×10^-2^ | 1.21 |
| Carbamic acid | 1.1×10^-2^ | 1.29 | 3.7×10^-1^ | 1.10 | 4.0×10^-2^ | 0.85 |
| Carnitine | 6.5×10^-1^ | 1.03 | 7.3×10^-4^ | 1.22 | 6.1×10^-3^ | 1.19 |
| Carnosine | 1.4×10^-1^ | 1.23 | 3.6×10^-1^ | 1.15 | 6.6×10^-1^ | 0.94 |
| Cholestenol | 3.9×10^-3^ | 1.29 | 1.8×10^-1^ | 1.13 | 6.1×10^-2^ | 0.88 |
| Cholesterol | 2.8×10^-1^ | 0.87 | 3.4×10^-1^ | 1.12 | 3.8×10^-2^ | 1.29 |
| Citrulline | 2.5×10^-1^ | 1.18 | 7.0×10^-2^ | 0.75 | 5.3×10^-3^ | 0.64 |
| Coumaric acid | 5.3×10^-2^ | 0.90 | 4.0×10^-6^ | 0.76 | 8.6×10^-3^ | 0.84 |
| Creatine | 5.5×10^-3^ | 0.90 | 1.2×10^-4^ | 0.86 | 2.5×10^-1^ | 0.96 |
| Creatinine | 1.4×10^-1^ | 0.96 | 9.5×10^-1^ | 1.00 | 1.4×10^-1^ | 1.04 |
| Csytathionine | 2.1×10^-5^ | 2.19 | 2.2×10^-9^ | 3.52 | 1.5×10^-3^ | 1.61 |
| Cysteine | 3.1×10^-1^ | 1.07 | 9.0×10^-3^ | 0.82 | 1.3×10^-3^ | 0.77 |
| Cytidine | 5.4×10^-3^ | 0.54 | 3.8×10^-4^ | 0.40 | 1.4×10^-1^ | 0.75 |
| Cytosine | 9.5×10^-2^ | 1.21 | 4.4×10^-4^ | 1.43 | 9.4×10^-2^ | 1.18 |
| Dehydroascorbic acid | 4.0×10^-3^ | 0.36 | 1.2×10^-3^ | 0.28 | 4.1×10^-1^ | 0.80 |
| Deoxyflurouridine | 9.9×10^-1^ | 1.00 | 8.8×10^-1^ | 0.98 | 7.9×10^-1^ | 0.98 |
| Deoxy-Ribose | 5.1×10^-1^ | 0.92 | 4.1×10^-1^ | 0.90 | 8.7×10^-1^ | 0.98 |
| Dimethylglycine | 4.2×10^-1^ | 1.05 | 9.5×10^-1^ | 1.00 | 3.7×10^-1^ | 0.95 |
| Docosahexanoic acid | 1.1×10^-2^ | 1.26 | 3.7×10^-1^ | 1.09 | 3.4×10^-2^ | 0.86 |
| Dopamine | 2.5×10^-1^ | 1.10 | 1.6×10^-1^ | 1.11 | 8.9×10^-1^ | 1.01 |
| Eicosapentaenoic acid | 3.4×10^-1^ | 1.57 | 8.6×10^-2^ | 2.06 | 5.2×10^-1^ | 1.30 |
| Fumaric acid | 2.7×10^-3^ | 1.15 | 4.4×10^-7^ | 1.25 | 5.3×10^-2^ | 1.09 |
| GABA | 2.7×10^-3^ | 1.20 | 5.3×10^-1^ | 1.04 | 2.4×10^-2^ | 0.87 |
| Galactofuranose | 9.2×10^-2^ | 0.88 | 7.6×10^-1^ | 0.98 | 1.5×10^-1^ | 1.11 |
| Gluconic acid | 5.6×10^-3^ | 1.38 | 1.4×10^-3^ | 1.43 | 7.3×10^-1^ | 1.03 |
| Glucose | 3.7×10^-4^ | 1.29 | 2.3×10^-2^ | 1.20 | 3.5×10^-1^ | 0.93 |
| Glutamate | 7.5×10^-5^ | 1.13 | 4.5×10^-3^ | 1.08 | 6.8×10^-2^ | 0.95 |
| Glutamine | 7.0×10^-1^ | 1.02 | 9.7×10^-1^ | 1.00 | 7.4×10^-1^ | 0.98 |
| Glutathione | 4.1×10^-1^ | 1.05 | 5.4×10^-4^ | 1.22 | 1.1×10^-2^ | 1.16 |
| Glycine | 2.4×10^-1^ | 0.93 | 2.5×10^-8^ | 0.68 | 4.7×10^-5^ | 0.74 |
| Guanidobutanoate | 3.2×10^-2^ | 0.87 | 3.5×10^-2^ | 0.89 | 7.8×10^-1^ | 1.02 |
| Guanine | 8.4×10^-3^ | 1.26 | 2.9×10^-4^ | 1.35 | 3.4×10^-1^ | 1.08 |
| Guanosine | 7.1×10^-2^ | 1.21 | 9.9×10^-3^ | 1.33 | 3.2×10^-1^ | 1.10 |
| Hexanedioic acid | 8.1×10^-3^ | 1.26 | 3.0×10^-1^ | 1.10 | 4.0×10^-2^ | 0.87 |
| Histidine | 1.6×10^-1^ | 0.91 | 7.7×10^-6^ | 0.74 | 8.2×10^-3^ | 0.81 |
| Homocysteine | 1.1×10^-3^ | 0.65 | 9.2×10^-3^ | 0.72 | 1.1×10^-1^ | 1.12 |
| Hydroxyanthranillate | 8.0×10^-1^ | 1.02 | 2.5×10^-2^ | 1.23 | 3.3×10^-2^ | 1.21 |
| Hydroxyguanine | 8.6×10^-1^ | 0.91 | 9.6×10^-1^ | 0.97 | 9.1×10^-1^ | 1.06 |
| Hypoxanthine | 4.5×10^-2^ | 1.07 | 2.5×10^-2^ | 0.93 | 7.9×10^-5^ | 0.87 |
| Indoleacetic acid | 1.5×10^-2^ | 1.14 | 1.2×10^-8^ | 1.34 | 1.4×10^-3^ | 1.18 |
| Inosine | 1.7×10^-5^ | 1.31 | 1.3×10^-7^ | 1.41 | 1.4×10^-1^ | 1.08 |
| Kynurenic acid | 6.3×10^-3^ | 1.49 | 7.3×10^-7^ | 2.03 | 8.3×10^-3^ | 1.37 |
| Lactic acid | 5.0×10^-2^ | 1.07 | 4.4×10^-1^ | 1.03 | 2.0×10^-1^ | 0.96 |
| L-DOPA | 5.5×10^-1^ | 1.03 | 7.5×10^-1^ | 0.98 | 3.5×10^-1^ | 0.95 |
| Leucine/Isoleucine | 4.6×10^-1^ | 0.96 | 5.8×10^-5^ | 0.77 | 2.1×10^-4^ | 0.80 |
| Linoleic acid | 8.1×10^-1^ | 0.97 | 2.0×10^-1^ | 1.17 | 1.3×10^-1^ | 1.21 |
| Linolenic acid | 8.2×10^-2^ | 1.10 | 8.7×10^-3^ | 1.17 | 1.7×10^-1^ | 1.06 |
| Lysine | 4.2×10^-3^ | 0.80 | 2.6×10^-7^ | 0.62 | 2.8×10^-3^ | 0.78 |
| Malate | 3.6×10^-2^ | 1.14 | 1.2×10^-1^ | 0.87 | 6.7×10^-4^ | 0.76 |
| Methionine | 6.8×10^-1^ | 1.04 | 1.6×10^-2^ | 0.76 | 1.8×10^-3^ | 0.73 |
| Methyl-aspartic acid | 2.4×10^-13^ | 0.62 | 4.7×10^-5^ | 0.81 | 2.9×10^-7^ | 1.32 |
| Methylheptadecadiynoate | 4.6×10^-1^ | 0.92 | 3.3×10^-1^ | 1.11 | 9.8×10^-2^ | 1.20 |
| Methylstearate | 1.9×10^-3^ | 0.41 | 5.9×10^-3^ | 0.47 | 6.4×10^-1^ | 1.15 |
| Nicotinamide | 1.8×10^-4^ | 1.14 | 4.7×10^-3^ | 1.12 | 5.5×10^-1^ | 0.98 |
| Nicotinic acid | 4.9×10^-3^ | 1.30 | 9.8×10^-1^ | 1.00 | 1.4×10^-2^ | 0.77 |
| Nitrotyrosine | 6.8×10^-1^ | 0.97 | 1.3×10^-4^ | 0.72 | 1.7×10^-3^ | 0.74 |
| Noradrenaline | 1.1×10^-2^ | 1.32 | 8.0×10^-1^ | 1.03 | 4.8×10^-2^ | 0.78 |
| Octadecanal | 1.1×10^-2^ | 0.13 | 6.7×10^-2^ | 0.32 | 2.4×10^-1^ | 2.44 |
| Oleic acid | 5.9×10^-1^ | 0.89 | 1.6×10^-1^ | 1.30 | 4.2×10^-2^ | 1.47 |
| Ornithine | 5.8×10^-2^ | 0.87 | 4.5×10^-6^ | 0.67 | 5.4×10^-3^ | 0.78 |
| Oxoarginine | 4.8×10^-1^ | 0.93 | 2.8×10^-1^ | 1.12 | 3.9×10^-2^ | 1.20 |
| Oxoglutarate | 8.8×10^-1^ | 0.99 | 3.6×10^-3^ | 1.17 | 5.4×10^-3^ | 1.18 |
| Palmitic acid | 7.9×10^-1^ | 0.96 | 1.8×10^-1^ | 1.22 | 1.1×10^-1^ | 1.28 |
| Pantothenate | 3.1×10^-1^ | 0.88 | 9.0×10^-2^ | 0.80 | 4.0×10^-1^ | 0.91 |
| Phenylalanine | 9.9×10^-1^ | 1.00 | 1.6×10^-3^ | 0.79 | 3.9×10^-4^ | 0.79 |
| Phosphocholine | 3.9×10^-1^ | 0.96 | 8.0×10^-5^ | 0.81 | 5.0×10^-3^ | 0.84 |
| Phosphocreatine | 1.4×10^-1^ | 0.78 | 5.3×10^-1^ | 1.10 | 2.8×10^-2^ | 1.42 |
| Proline | 8.8×10^-2^ | 0.94 | 4.9×10^-8^ | 0.77 | 4.2×10^-5^ | 0.82 |
| Propionylcarnitine | 7.5×10^-7^ | 0.46 | 2.9×10^-6^ | 0.49 | 5.3×10^-1^ | 1.07 |
| Serine | 4.8×10^-1^ | 0.93 | 4.5×10^-2^ | 0.79 | 8.0×10^-2^ | 0.85 |
| Spermidine | 5.0×10^-1^ | 1.17 | 5.1×10^-1^ | 0.86 | 1.9×10^-1^ | 0.74 |
| Succinate | 2.9×10^-1^ | 0.92 | 1.8×10^-1^ | 1.10 | 2.1×10^-2^ | 1.20 |
| Taurine | 1.7×10^-6^ | 0.74 | 1.2×10^-5^ | 0.77 | 3.9×10^-1^ | 1.04 |
| Threonine | 1.6×10^-1^ | 0.93 | 1.0×10^-6^ | 0.74 | 3.2×10^-4^ | 0.79 |
| Tryptophan | 3.4×10^-1^ | 1.10 | 6.1×10^-2^ | 0.80 | 2.1×10^-3^ | 0.73 |
| Tyrosine | 4.6×10^-1^ | 0.94 | 3.6×10^-4^ | 0.72 | 2.0×10^-3^ | 0.76 |
| Uracil | 6.9×10^-1^ | 0.97 | 4.1×10^-2^ | 0.87 | 4.7×10^-2^ | 0.89 |
| Uric acid | 7.9×10^-1^ | 1.03 | 4.5×10^-1^ | 0.91 | 2.3×10^-1^ | 0.88 |
| Uridine | 2.4×10^-13^ | 0.53 | 9.6×10^-6^ | 0.75 | 7.2×10^-7^ | 1.40 |
| Valine | 1.2×10^-1^ | 0.89 | 7.0×10^-6^ | 0.68 | 3.9×10^-3^ | 0.77 |
| Xanthine | 2.8×10^-8^ | 1.25 | 8.9×10^-4^ | 1.13 | 3.2×10^-4^ | 0.91 |
| Xanthosine | 2.8×10^-2^ | 1.18 | 3.3×10^-1^ | 1.07 | 1.6×10^-1^ | 0.91 |

^a^ p-value calculated using mann-whitney U-test. ^b^ fold change relative to cerebellum, ^c^ fold change relative to inferior temporal gyrus. AMP; adenosine-monophosphate, CB; cerebellum, GABA; gamma-aminobutanoate, ITG; inferior temporal gyrus, L-DOPA; L-dihydroxy-phenylalanine, MFG; medial frontal gyrus.
