# Supplementary material for: Association between fatty acid metabolism in the brain and Alzheimer disease neuropathology and cognitive performance: A nontargeted metabolomic study
Source: PLoS Med. 2017 Mar 21;14(3):e1002266. doi: 10.1371/journal.pmed.1002266 (PMC5360226; doi:10.1371/journal.pmed.1002266)
Supplement: S3 Table — Relationships between global measures of cross sectional and longitudinal memory performance and the regional abundances of six UFAs, values highlighted in bold are significant at p < 0.05. * correlation of fatty acid abundance to last memory score before death, + correlation of fatty acid abundance to rate of longitudinal decline in memory. CERAD; Consortium to Establish a Registry for Alzheimer’s Disease. (DOCX) [file pmed.1002266.s004.docx]

**S3 Table Correlation of the abundance of 6 unsaturated fatty acids with measures of both cross sectional and longitudinal memory performance.**

|  |  | **Last Score^*^** | | **Longitudinal decline^+^** | |
| --- | --- | --- | --- | --- | --- |
|  |  | **Estimate** | **p-value** | **Estimate** | **p-value** |
| **CB** | **Eicosapentaenoic acid** | 0.185 | 0.551 | 0.020 | 0.490 |
|  | **Linoleic acid** | 0.042 | 0.867 | 0.019 | 0.394 |
|  | **Arachidonic acid** | 0.275 | 0.264 | 0.039 | 0.093 |
|  | **Oleic acid** | **0.489** | **0.032** | **0.047** | **0.029** |
|  | **Docosahexanoic acid** | **-0.607** | **0.022** | **-0.052** | **0.035** |
|  | **Linolenic acid** | 0.305 | 0.209 | 0.023 | 0.290 |
| **ITG** | **Eicosapentaenoic acid** | 0.073 | 0.731 | 0.000 | 0.988 |
|  | **Linoleic acid** | 0.238 | 0.231 | 0.010 | 0.599 |
|  | **Arachidonic acid** | 0.236 | 0.244 | 0.011 | 0.585 |
|  | **Oleic acid** | 0.182 | 0.373 | 0.006 | 0.751 |
|  | **Docosahexanoic acid** | -0.131 | 0.528 | 0.003 | 0.866 |
|  | **Linolenic acid** | 0.282 | 0.188 | 0.014 | 0.515 |
| **MFG** | **Eicosapentaenoic acid** | 0.109 | 0.620 | 0.027 | 0.206 |
|  | **Linoleic acid** | 0.344 | 0.121 | 0.039 | 0.075 |
|  | **Arachidonic acid** | 0.283 | 0.200 | 0.034 | 0.116 |
|  | **Oleic acid** | 0.350 | 0.121 | 0.039 | 0.080 |
|  | **Docosahexanoic acid** | -0.347 | 0.148 | -0.038 | 0.105 |
|  | **Linolenic acid** | 0.268 | 0.249 | 0.034 | 0.139 |

Relationships between global measures of cross sectional and longitudinal memory performance and the regional abundances of 6 unsaturated fatty acids, values highlighted in bold are significant at p<0.05. ^*^ correlation of fatty acid abundance to last memory score before death, ^+^ correlation of fatty acid abundance to rate of longitudinal decline in memory.
