# Supplementary material for: Association between fatty acid metabolism in the brain and Alzheimer disease neuropathology and cognitive performance: A nontargeted metabolomic study
Source: PLoS Med. 2017 Mar 21;14(3):e1002266. doi: 10.1371/journal.pmed.1002266 (PMC5360226; doi:10.1371/journal.pmed.1002266)
Supplement: S4 Table — Relationships between global measures of cross sectional and longitudinal language performance and the regional abundances of six UFAs, values highlighted in bold are significant at p < 0.05. * correlation of fatty acid abundance to last language score before death, + correlation of fatty acid abundance to rate of longitudinal decline in language. CERAD; Consortium to Establish a Registry for Alzheimer’s Disease. (DOCX) [file pmed.1002266.s005.docx]

**S4 Table Correlation of the abundance of 6 unsaturated fatty acids with measures of both cross sectional and longitudinal language performance.**

|  |  | **Last Score^*^** | | **Longitudinal decline^+^** | |
| --- | --- | --- | --- | --- | --- |
|  |  | **Estimate** | **p-value** | **Estimate** | **p-value** |
| **CB** | **Eicosapentaenoic acid** | 0.068 | 0.804 | 0.015 | 0.583 |
|  | **Linoleic acid** | 0.176 | 0.404 | 0.008 | 0.644 |
|  | **Arachidonic acid** | 0.154 | 0.472 | 0.017 | 0.347 |
|  | **Oleic acid** | 0.194 | 0.346 | 0.018 | 0.273 |
|  | **Docosahexanoic acid** | -0.053 | 0.817 | -0.020 | 0.272 |
|  | **Linolenic acid** | 0.294 | 0.154 | 0.020 | 0.225 |
| **ITG** | **Eicosapentaenoic acid** | 0.253 | 0.178 | -0.002 | 0.907 |
|  | **Linoleic acid** | 0.371 | 0.050 | 0.026 | 0.127 |
|  | **Arachidonic acid** | 0.348 | 0.067 | 0.025 | 0.156 |
|  | **Oleic acid** | 0.341 | 0.077 | 0.023 | 0.207 |
|  | **Docosahexanoic acid** | **-0.384** | **0.044** | -0.017 | 0.354 |
|  | **Linolenic acid** | 0.329 | 0.074 | 0.023 | 0.217 |
| **MFG** | **Eicosapentaenoic acid** | 0.105 | 0.605 | **0.054** | **0.032** |
|  | **Linoleic acid** | 0.230 | 0.253 | **0.047** | **0.015** |
|  | **Arachidonic acid** | 0.173 | 0.383 | **0.043** | **0.026** |
|  | **Oleic acid** | 0.236 | 0.215 | **0.047** | **0.012** |
|  | **Docosahexanoic acid** | -0.125 | 0.499 | -0.033 | 0.074 |
|  | **Linolenic acid** | 0.229 | 0.242 | **0.046** | **0.015** |

Relationships between global measures of cross sectional and longitudinal language performance and the regional abundances of 6 unsaturated fatty acids, values highlighted in bold are significant at p<0.05. ^*^ correlation of fatty acid abundance to last language score before death, ^+^ correlation of fatty acid abundance to rate of longitudinal decline in language.
