# Supplementary material for: Association between fatty acid metabolism in the brain and Alzheimer disease neuropathology and cognitive performance: A nontargeted metabolomic study
Source: PLoS Med. 2017 Mar 21;14(3):e1002266. doi: 10.1371/journal.pmed.1002266 (PMC5360226; doi:10.1371/journal.pmed.1002266)
Supplement: S5 Table — Relationships between global measures of cross sectional and longitudinal attention span performance and the regional abundances of six UFAs, values highlighted in bold are significant at p < 0.05. * correlation of fatty acid abundance to last attention span score before death, + correlation of fatty acid abundance to rate of longitudinal decline in attention span. CERAD; Consortium to Establish a Registry for Alzheimer’s Disease. (DOCX) [file pmed.1002266.s006.docx]

**S5 Table Correlation of the abundance of 6 unsaturated fatty acids with measures of both cross sectional and longitudinal attention span.**

|  |  | **Last Score^*^** | | **Longitudinal decline^+^** | |
| --- | --- | --- | --- | --- | --- |
|  |  | **Estimate** | **p-value** | **Estimate** | **p-value** |
| **CB** | **Eicosapentaenoic acid** | 0.152 | 0.650 | -0.027 | 0.382 |
|  | **Linoleic acid** | 0.222 | 0.234 | 0.016 | 0.384 |
|  | **Arachidonic acid** | 0.220 | 0.244 | 0.016 | 0.392 |
|  | **Oleic acid** | 0.306 | 0.058 | 0.031 | 0.003 |
|  | **Docosahexanoic acid** | -0.065 | 0.755 | -0.011 | 0.555 |
|  | **Linolenic acid** | **0.356** | **0.031** | **0.026** | **0.003** |
| **ITG** | **Eicosapentaenoic acid** | 0.277 | 0.093 | 0.013 | 0.502 |
|  | **Linoleic acid** | 0.230 | 0.173 | 0.013 | 0.455 |
|  | **Arachidonic acid** | 0.202 | 0.236 | 0.013 | 0.494 |
|  | **Oleic acid** | 0.189 | 0.269 | 0.011 | 0.549 |
|  | **Docosahexanoic acid** | -0.256 | 0.123 | -0.009 | 0.631 |
|  | **Linolenic acid** | 0.284 | 0.102 | 0.014 | 0.475 |
| **MFG** | **Eicosapentaenoic acid** | 0.233 | 0.184 | 0.011 | 0.640 |
|  | **Linoleic acid** | **0.385** | **0.027** | 0.030 | 0.117 |
|  | **Arachidonic acid** | **0.347** | **0.042** | 0.028 | 0.138 |
|  | **Oleic acid** | **0.352** | **0.042** | 0.032 | 0.096 |
|  | **Docosahexanoic acid** | -0.345 | 0.064 | -0.034 | 0.087 |
|  | **Linolenic acid** | **0.387** | **0.031** | 0.027 | 0.173 |

Relationships between global measures of cross sectional and longitudinal attention span performance and the regional abundances of 6 unsaturated fatty acids, values highlighted in bold are significant at p<0.05. ^*^ correlation of fatty acid abundance to last attention span score before death, ^+^ correlation of fatty acid abundance to rate of longitudinal decline in attention span.
