# Supplementary material for: Association between fatty acid metabolism in the brain and Alzheimer disease neuropathology and cognitive performance: A nontargeted metabolomic study
Source: PLoS Med. 2017 Mar 21;14(3):e1002266. doi: 10.1371/journal.pmed.1002266 (PMC5360226; doi:10.1371/journal.pmed.1002266)
Supplement: S6 Table — Relationships between global measures of cross sectional and longitudinal attention span performance and the regional abundances of six UFAs, values highlighted in bold are significant at p < 0.05. * correlation of fatty acid abundance to last executive function score before death, + correlation of fatty acid abundance to rate of longitudinal decline in executive function score. CERAD; Consortium to Establish a Registry for Alzheimer’s Disease. (DOCX) [file pmed.1002266.s007.docx]

**S6 Table Correlation of the abundance of 6 unsaturated fatty acids with measures of both cross sectional and longitudinal executive function.**

|  |  | **Last Score^*^** | | **Longitudinal decline^+^** | |
| --- | --- | --- | --- | --- | --- |
|  |  | **Estimate** | **p-value** | **Estimate** | **p-value** |
| **CB** | **Eicosapentaenoic acid** | -0.007 | 0.984 | 0.005 | 0.765 |
|  | **Linoleic acid** | 0.119 | 0.552 | 0.008 | 0.396 |
|  | **Arachidonic acid** | 0.193 | 0.336 | 0.011 | 0.288 |
|  | **Oleic acid** | 0.349 | 0.068 | 0.016 | 0.126 |
|  | **Docosahexanoic acid** | -0.258 | 0.227 | -0.009 | 0.476 |
|  | **Linolenic acid** | 0.376 | 0.053 | 0.008 | 0.383 |
| **ITG** | **Eicosapentaenoic acid** | 0.244 | 0.160 | -0.004 | 0.764 |
|  | **Linoleic acid** | 0.273 | 0.123 | 0.003 | 0.768 |
|  | **Arachidonic acid** | 0.248 | 0.165 | 0.002 | 0.855 |
|  | **Oleic acid** | 0.262 | 0.145 | 0.004 | 0.742 |
|  | **Docosahexanoic acid** | **-0.357** | **0.038** | -0.006 | 0.620 |
|  | **Linolenic acid** | **0.379** | **0.037** | 0.011 | 0.400 |
| **MFG** | **Eicosapentaenoic acid** | 0.270 | 0.134 | 0.005 | 0.775 |
|  | **Linoleic acid** | **0.525** | **0.002** | 0.008 | 0.545 |
|  | **Arachidonic acid** | **0.484** | **0.005** | 0.009 | 0.468 |
|  | **Oleic acid** | **0.518** | **0.003** | 0.009 | 0.477 |
|  | **Docosahexanoic acid** | **-0.575** | **0.002** | -0.007 | 0.654 |
|  | **Linolenic acid** | **0.550** | **0.002** | 0.006 | 0.670 |

Relationships between global measures of cross sectional and longitudinal attention span performance and the regional abundances of 6 unsaturated fatty acids, values highlighted in bold are significant at p<0.05. ^*^ correlation of fatty acid abundance to last executive function score before death, ^+^ correlation of fatty acid abundance to rate of longitudinal decline in executive function score.
