# Supplementary material for: Association between fatty acid metabolism in the brain and Alzheimer disease neuropathology and cognitive performance: A nontargeted metabolomic study
Source: PLoS Med. 2017 Mar 21;14(3):e1002266. doi: 10.1371/journal.pmed.1002266 (PMC5360226; doi:10.1371/journal.pmed.1002266)
Supplement: S7 Table — Relationships between global measures of cross sectional and longitudinal attention span performance and the regional abundances of six UFAs, values highlighted in bold are significant at p < 0.05. * correlation of fatty acid abundance to last visual spatial awareness score before death, + correlation of fatty acid abundance to rate of longitudinal decline in visual spatial awareness score. CERAD; Consortium to Establish a Registry for Alzheimer’s Disease. (DOCX) [file pmed.1002266.s008.docx]

**S7 Table Correlation of the abundance of 6 unsaturated fatty acids with measures of both cross sectional and longitudinal visual spatial awareness.**

|  |  | **Last Score^*^** | | **Longitudinal decline^+^** | |
| --- | --- | --- | --- | --- | --- |
|  |  | **Estimate** | **p-value** | **Estimate** | **p-value** |
| **CB** | **Eicosapentaenoic acid** | 0.383 | 0.166 | **0.055** | **0.013** |
|  | **Linoleic acid** | 0.296 | 0.091 | 0.025 | 0.218 |
|  | **Arachidonic acid** | **0.349** | **0.020** | **0.033** | **0.015** |
|  | **Oleic acid** | **0.422** | **0.004** | **0.042** | **0.002** |
|  | **Docosahexanoic acid** | -0.182 | 0.366 | -0.025 | 0.248 |
|  | **Linolenic acid** | **0.329** | **0.033** | **0.035** | **0.004** |
| **ITG** | **Eicosapentaenoic acid** | 0.038 | 0.815 | 0.030 | 0.079 |
|  | **Linoleic acid** | 0.160 | 0.317 | **0.029** | **0.034** |
|  | **Arachidonic acid** | 0.131 | 0.417 | 0.028 | 0.056 |
|  | **Oleic acid** | 0.128 | 0.434 | **0.032** | **0.027** |
|  | **Docosahexanoic acid** | -0.218 | 0.173 | **-0.033** | **0.014** |
|  | **Linolenic acid** | 0.266 | 0.113 | **0.042** | **0.008** |
| **MFG** | **Eicosapentaenoic acid** | 0.258 | 0.118 | 0.025 | 0.186 |
|  | **Linoleic acid** | **0.330** | **0.043** | 0.025 | 0.094 |
|  | **Arachidonic acid** | 0.305 | 0.059 | 0.026 | 0.084 |
|  | **Oleic acid** | 0.303 | 0.065 | 0.028 | 0.065 |
|  | **Docosahexanoic acid** | -0.235 | 0.176 | -0.031 | 0.074 |
|  | **Linolenic acid** | 0.298 | 0.076 | 0.023 | 0.144 |

Relationships between global measures of cross sectional and longitudinal attention span performance and the regional abundances of 6 unsaturated fatty acids, values highlighted in bold are significant at p<0.05. ^*^ correlation of fatty acid abundance to last visual spatial awareness score before death, ^+^ correlation of fatty acid abundance to rate of longitudinal decline in visual spatial awareness score.
