# Supplementary figures and images for: Association between fatty acid metabolism in the brain and Alzheimer disease neuropathology and cognitive performance: A nontargeted metabolomic study
Source: PLoS Med. 2017 Mar 21;14(3):e1002266. doi: 10.1371/journal.pmed.1002266 (PMC5360226; doi:10.1371/journal.pmed.1002266)

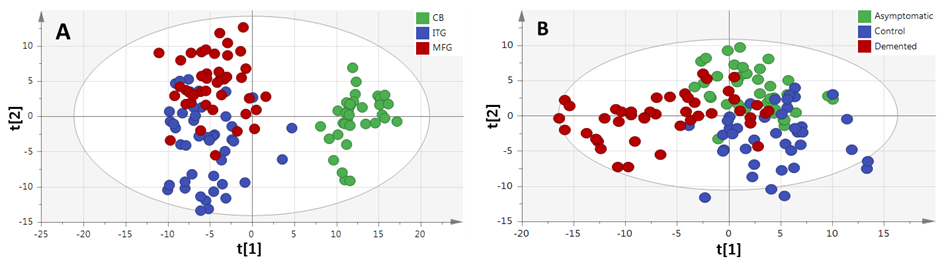

Supplement: S1 Fig — A) Comparison of metabolite composition of CB, ITG, and MFG (R2X = 0.639, R2Y = 0.622, Q2 = 0.608, CV-ANOVA = 5.65 × 10^−33^) B) Comparison of metabolite composition of diagnostic groups, control, asymptomatic, and individuals with dementia (R2X = 0.499, R2Y = 0.439, Q2 = 0.404, CV-ANOVA = 4.72 × 10^−21^). (TIF) [file pmed.1002266.s009.tif]

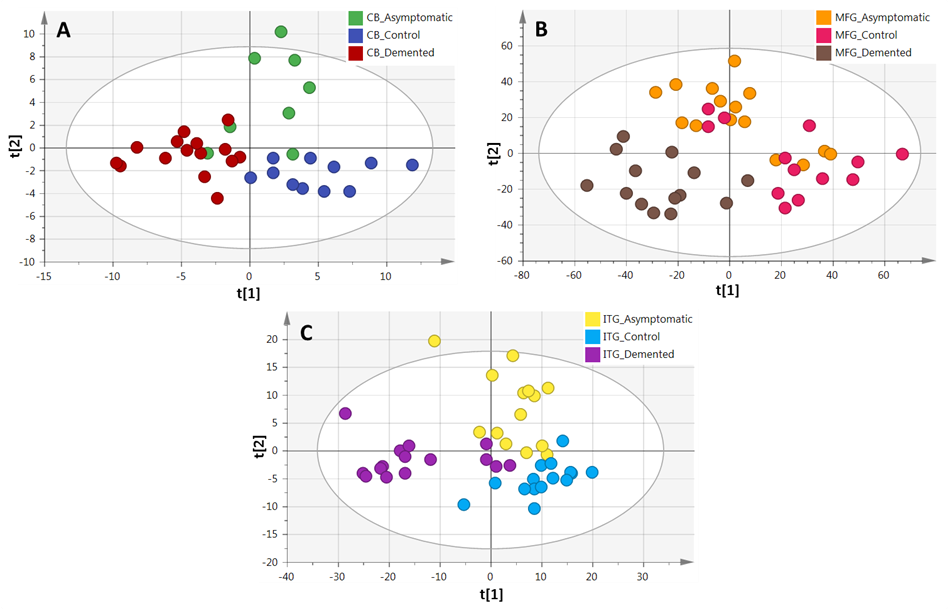

Supplement: S2 Fig — A) Comparison of metabolite composition of diagnostic groups, control, asymptomatic, and individuals with dementia in CB samples (R2X = 0.527, R2Y = 0.539, Q2 = 0.424, CV-ANOVA = 5.04 × 10^−4^), B) Comparison of metabolite composition of diagnostic groups, control, asymptomatic, and individuals with dementia in ITG samples (R2X = 0.592, R2Y = 0.563, Q2 = 0.424, CV-ANOVA = 3.28 × 10^−7^), C) Comparison of metabolite composition of diagnostic groups, control, asymptomatic, and individual with dementia in MFG samples (R2X = 0.650, R2Y = 0.563, Q2 = 0.490, CV-ANOVA = 1.62 × 10^−5^). (TIF) [file pmed.1002266.s010.tif]

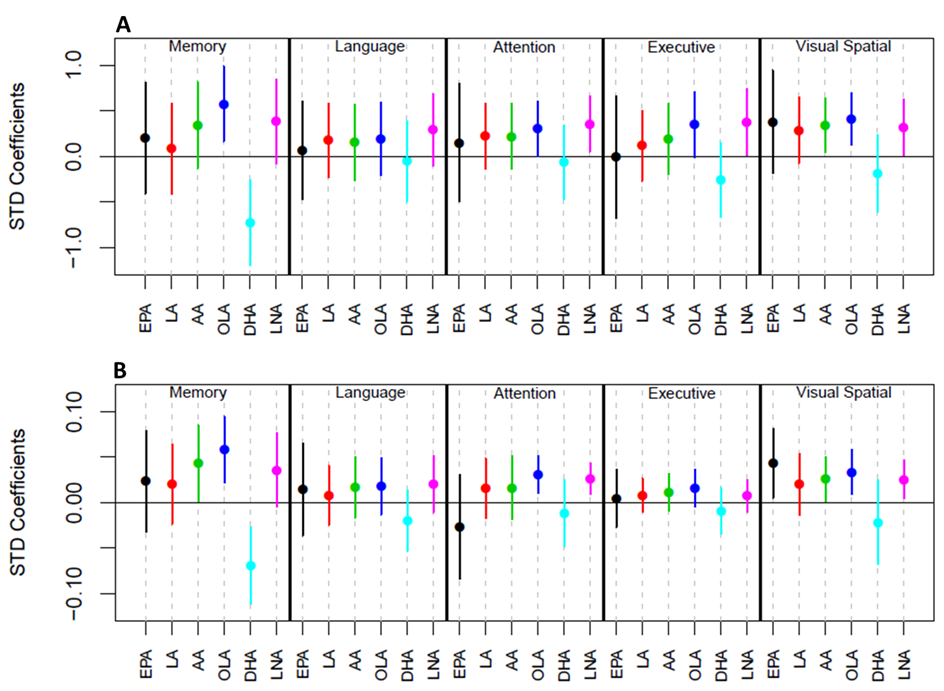

Supplement: S3 Fig — A) association between fatty acid abundance of UFA levels and longitudinal cognitive performance B) associations between fatty acid abundance cognitive performance at the last visit prior to death. AA: arachidonic acid, DHA: docosahexaenoic acid, EPA: eicosapentaenoic acid, LA: linoleic acid, LNA: linolenic acid, OLA: oleic acid. (TIF) [file pmed.1002266.s011.tif]

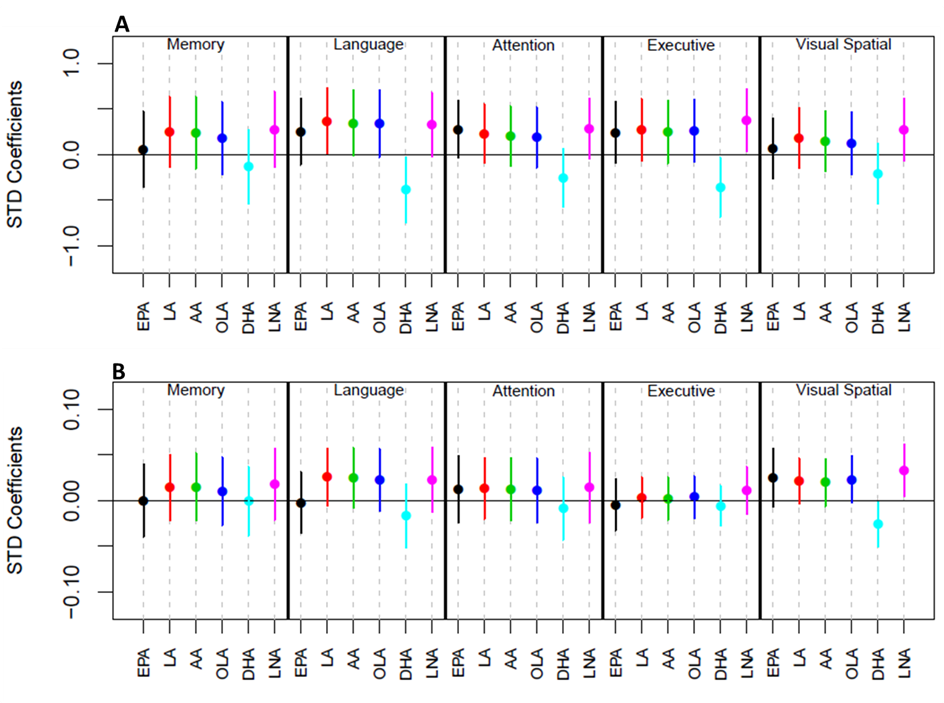

Supplement: S4 Fig — A) association between fatty acid abundance of UFA levels and longitudinal cognitive performance B) associations between fatty acid abundance cognitive performance at the last visit prior to death. AA: arachidonic acid, DHA: docosahexaenoic acid, EPA: eicosapentaenoic acid, LA: linoleic acid, LNA: linolenic acid, OLA: oleic acid. (TIF) [file pmed.1002266.s012.tif]
