# Supplementary material for: Association between fatty acid metabolism in the brain and Alzheimer disease neuropathology and cognitive performance: A nontargeted metabolomic study
Source: PLoS Med. 2017 Mar 21;14(3):e1002266. doi: 10.1371/journal.pmed.1002266 (PMC5360226; doi:10.1371/journal.pmed.1002266)
Supplement: S1 Text — (DOCX) [file pmed.1002266.s013.docx]

**Supplemental Analysis Plan**

In this study we planned to apply comprehensive metabolic profiling techniques to the sample cohort and to combine the generated data with the extensive cognitive information available for these samples. As with all metabolomics studies the analyses that we performed following an in-house pipeline in which some steps are ‘data driven’ with the outcome of one analysis informing the next, the manuscript contains an extensive and detailed section describing the statistical workflow applied used in this study, and is summarised below.

**General pipeline for metabolomics data analysis**

1. Raw LC-MS and GC-MS data were processed by first converting instrument outputs to .MZxml files before identifying peaks using XCMS a standard approached that is widely used in the metabolomics field, with generated data normalised to total ion count.
2. Initial metabolite selection was performed using principal component analysis (PCA) and partial least square – discriminant analysis (PLS-DA) performed in SIMCA 13.0.4 (Umetrics, Umeå, Sweden).
3. After data dependent identification of the fatty acids we compared these metabolites among 3 groups (CN, ASYMAD and DEMENT), using nonparametric Kruskal–Wallis test for testing the null hypothesis that all 3 groups are equal and Mann–Whitney U test for pairwise comparisons.
4. To control for type 1 errors we applied both Benjamini-Hochberg correction and the stringent Bonferroni corrected significance threshold.
5. Having shown associations with diagnostic status, we used Pearson’s product-moment correlation to identify associations to neuritic plaque and neurofibrillary tangle burdens.
6. The final stage of the analysis was to evaluate the relationship between the abundance of these fatty acids and longitudinal cognitive performance. To do this we created domain-specific composite scores, we standardized each of the cognitive measures (using their baseline mean and standard deviation) and averaged the corresponding Z scores to form the composite score. We used separate linear mixed models with each cognitive domain as the outcome variable. The main predictor time (or time of follow up) was anchored at the last visit, and all the previous longitudinal follow-up visits were negative relative to the last visit. The predictors included fatty acid levels, sex, age at last visit, time and interactions of time with fatty acid levels, sex and age at last visit.
